# PET-Fe-BDC Composite Geotextiles for Groundwater Remediation: In Situ Synthesis and Dynamic Retention of Pb(II) and Cd(II)

**DOI:** 10.3390/ma19194100

**Published:** 2026-09-25

**Authors:** Li Shao, Kang Wu, Jing Ni, Jiawei Xu, Tongtong Wang, Jili Qu, Chao Bao

**Affiliations:** 1 School of Environment and Architecture, University of Shanghai for Science and Technology, Shanghai 200093, China; 2 SGIDI Engineering Consulting (Group) Co., Ltd., Shanghai 200093, China; 3 School of Civil Engineering, Kashi University, Kashi 844008, China

**Keywords:** Fe-BDC, geotextile composites, dynamic retention, groundwater remediation, breakthrough modeling

## Abstract

Remediating heavy-metal pollution in flowing groundwater is challenging because powder adsorbents may agglomerate or wash away, while static tests do not represent dynamic transport. To address these limitations, a PET-Fe-BDC composite geotextile was prepared by in situ synthesis following PAM pretreatment and evaluated for Pb(II) and Cd(II) retention in continuous soil-column seepage experiments. Compared with unamended soil, the composite-geotextile-amended system markedly retarded the downward migration of both ions. In the 0–5 cm retardation layer, cumulative removal efficiencies exceeded 80% up to *t_e_*′, and the breakthrough times of Pb(II) and Cd(II) increased to approximately 6.9 and 6.4 times those in the unamended system, respectively. Thomas, Yan, Clark, and Yoon–Nelson models were used to describe the observed breakthrough behavior. The Yan model provided the most consistent empirical description across the tested curves. The Thomas model closely described the unamended-soil curves and the composite-system Cd(II) curve but showed weaker agreement for composite-system Pb(II), whereas the Clark and Yoon–Nelson models showed lower fitting performance. Because complete saturation was not reached, model-derived parameters were interpreted comparatively. Overall, the integrated PET-Fe-BDC composite-geotextile-amended soil system substantially delayed Pb(II) and Cd(II) breakthrough under continuous seepage, demonstrating its potential for groundwater-remediation barriers.

## 1. Introduction

With the rapid advancement of industrialization, wastewater generated by metal smelting, mining, electroplating, and related activities has become an important source of heavy-metal contamination in soil and groundwater. Field investigations in representative Pb–Zn mining and smelting regions in China have documented elevated concentrations of Cd, Pb, Zn, As, and Sb in surrounding environmental media [1,2,3]. Among these contaminants, Pb(II) and Cd(II) are of particular concern because of their persistence, non-biodegradability, and potential to migrate with groundwater flow and accumulate in environmental and biological systems [4,5].

To effectively curb the dissemination of groundwater pollution, the permeable reactive barrier (PRB)—an in situ remediation technology—has emerged as a frontier in groundwater heavy metal treatment, owing to its zero external power requirement, high treatment efficiency, and minimal surface disturbance [6,7,8]. The efficacy of PRB technology fundamentally depends on the selection of reactive filling materials [9,10,11,12]. However, traditional physical adsorbents, such as activated carbon and zeolite, generally suffer from intrinsic bottlenecks, including limited adsorption capacity, poor selectivity, and premature breakthrough [13,14].

In recent years, metal–organic frameworks (MOFs) have received considerable attention in water treatment because of their tunable coordination environments and surface functional groups [14,15,16,17]. Recent reviews have summarized the development of Fe-based MOFs and MOF composites for the removal of heavy metals from water [18,19,20]. Research has also advanced into dynamic flow configurations. Representative examples include Fe- and Zr-based MOFs immobilized in electrospun polymer nanofibers for Pb(II) and Hg(II) filtration, Fe-BTC/polydopamine composite beads used for continuous Pb(II) removal, and Ca-MOF/cellulose beads evaluated in packed-bed columns for Pb(II), Cd(II), and Cu(II) retention [21,22,23]. These studies demonstrate the feasibility of structured MOF materials in flow-through treatment and highlight the importance of carrier geometry, hydraulic accessibility, and breakthrough behavior.

To facilitate the use of MOFs in flow-through systems, a variety of macroscopic configurations have been developed, including hierarchically porous adsorbents, monoliths, polymer membranes and nanofibers, hydrogel matrices, robust foams, mineral supports, and geopolymer fibers [24,25,26,27,28,29,30,31,32]. These configurations provide different combinations of hydraulic accessibility, mechanical integrity, and adsorbent retention. For subsurface barrier applications, a continuous sheet-like carrier is particularly attractive because it can be positioned across the groundwater flow path while avoiding the direct dispersion of fine adsorbent particles into the aquifer.

Needle-punched PET geotextiles provide an interconnected fibrous network, favorable cross-plane water permeability, mechanical handleability, and established compatibility with soil and drainage systems [33,34]. Compared with dispersed powders or densely packed adsorbent layers, the open nonwoven structure preserves interconnected water-flow pathways while providing a macroscopically continuous substrate for Fe-BDC deposition. Its sheet-like configuration also facilitates controlled installation, localized placement, and replacement during PRB maintenance. Moreover, PET geotextiles can be manufactured using recycled polyester fibers, providing a practical route for incorporating circular-material considerations into future engineering deployment [35]. These hydraulic, mechanical, and operational characteristics motivated the selection of PET geotextile as the supporting scaffold in this study.

In the preparation route, the activated PET geotextile was sequentially treated with PAM, exposed to Fe^3+^, and subjected to solvothermal synthesis. PAM-containing polymer/MOF interfaces have previously been employed in the construction of supported composite materials [36,37]. In this study, PAM was applied as an intermediate coating step before Fe^3+^ exposure and subsequent Fe-BDC synthesis. The resulting PET-Fe-BDC composite was characterized using PXRD, FTIR, XPS, and SEM before evaluation in the column seepage experiments.

Accordingly, this study evaluated the dynamic retention performance of the integrated PET-Fe-BDC composite-geotextile-amended soil system under continuous seepage conditions. The composite geotextile was prepared through PET activation, PAM pretreatment, Fe^3+^ preloading, and in situ solvothermal synthesis. The study objectives were to: (i) characterize the crystalline features, chemical bonding environments, surface composition, and morphology of the prepared material; (ii) quantify the depth- and time-dependent retention of Pb(II) and Cd(II) in continuous soil-column experiments; and (iii) compare the applicability of the Thomas, Yan, Clark, and Yoon–Nelson models for describing the observed breakthrough behavior. By combining a macroscopically deployable reactive geotextile with depth-resolved column monitoring, this work provides an engineering-oriented evaluation of heavy-metal retention and migration in the integrated soil–composite system under simulated groundwater seepage conditions.

## 2. Materials and Methods

### 2.1. Preparation of Experimental Materials

#### Selection of Materials

The soil samples used in this study were collected from a depth of 0–50 cm along the Fuxing River, a tributary of the Huangpu River. The samples were oven-dried at 105 °C for 24 h until reaching a constant weight, then pulverized and passed through a 0.05 mm sieve. The basic physicochemical properties of the soil are summarized in Table 1, and the particle size distribution curve is presented in Figure 1. The clay content (particle size < 0.005 mm) was determined to be 85.69%, classifying the soil as low-liquid-limit clay (CL). The polyester (PET) geotextile employed in the experiments had a mass per unit area of 300 g/m^2^. The key specifications and parameters of the PET geotextile are detailed in Table 2.

### 2.2. In Situ Synthesis of PET-Fe-BDC Composite Geotextiles

The preparation procedure comprised PET surface activation, PAM pretreatment, Fe^3+^ preloading, and subsequent in situ solvothermal synthesis of the Fe-BDC phase, as illustrated in Figure 2. All chemical reagents were purchased from Sinopharm Chemical Reagent Co., Ltd. (Shanghai, China) and used as received.

PET Activation: The PET geotextile was first cut into 5 cm × 8 cm pieces, washed three times with detergent, ethanol, and deionized water, respectively, and then dried at 60 °C until reaching a constant weight. The dried PET was immersed in a 4 mol/L NaOH solution and stirred continuously at 80 °C for 2 h. Subsequently, the samples were rinsed with a 1 mol/L HCl solution until neutrality, followed by thorough rinsing with deionized water and drying at 60 °C to constant weight to obtain the activated PET. The activated PET was then cut into 5 cm × 8 cm and 1 cm × 1 cm pieces, immersed in a 1% PAM solution for 12 h, and dried at 60 °C to constant weight to obtain the PAM-modified PET (PAM-PET).

Preparation of PET-Fe-BDC: As illustrated in Figure 2, Solution A was prepared by dissolving FeCl_3_ (0.162 g, 1.0 mmol) in 41 mL of N,N-dimethylformamide (DMF). The PAM-PET was immersed in Solution A for 1 h to obtain Fe^3+^-PAM-PET. Meanwhile, Solution B was prepared by dissolving H_2_BDC (0.166 g, 1.0 mmol) in 41 mL of DMF. Subsequently, Solutions A and B were thoroughly mixed. This mixture, along with the PAM-PET oriented vertically, was transferred into a 100 mL Teflon-lined stainless steel autoclave and reacted at 120 °C for 24 h. Upon completion, the reaction system was naturally cooled to room temperature. The resulting PET-Fe-BDC composite was washed three times with DMF and ethanol, respectively, dried at 60 °C to constant weight, and finally activated in a vacuum at 150 °C for 10 h for subsequent use. The autoclave and forced-air drying oven (DHG-9140A, Shanghai Yiheng Scientific Instrument Co., Ltd., Shanghai, China) used for the preparation of the PET-Fe-BDC composite are shown in Figure 3. The column apparatus is illustrated schematically in Figure 4, and photographs of the experimental setup are shown in Figure 5.

### 2.3. Characterization Methods

The crystalline characteristics of the independently synthesized Fe-BDC powder, activated PET, and PET-Fe-BDC composite were examined by powder X-ray diffraction (PXRD; Ultima IV, Rigaku Corporation, Tokyo, Japan) over a 2θ range of 5–90°. The diffraction pattern of the Fe-BDC powder was compared with ICDD PDF 33-1723 for iron terephthalate hydroxide, Fe(OH)(BDC). Fourier-transform infrared (FTIR; Nicolet iN10, Thermo Fisher Scientific, Waltham, MA, USA) spectra of Fe-BDC and PET-Fe-BDC were evaluated over the range of 4000–600 cm^−1^ to identify the characteristic functional groups of the iron–terephthalate phase. The surface elemental composition and chemical environments were examined by X-ray photoelectron spectroscopy (XPS; K-Alpha, Thermo Fisher Scientific, East Grinstead, UK), including survey spectra and high-resolution C 1s, O 1s, and Fe 2p spectra. The apparent gravimetric loading fraction was calculated as the net increase in dry mass of the final PET-Fe-BDC composite relative to the initial dry mass of the activated PET substrate: $L_{\mathrm{app}}=(m_{c}-m_{0})/m_{0}\times 100\%$, where *m*_0_ is the dry mass of the activated PET substrate before PAM pretreatment and subsequent solvothermal synthesis, and *m*_c_ is the dry mass of the corresponding PET-Fe-BDC composite after washing and drying to constant mass. The reported value was the mean of 3 separate preparation batches (*n* = 3). Because this value represents the net mass change over the full preparation sequence, including PAM pretreatment and possible PET mass loss, it is reported as an apparent semiquantitative loading fraction rather than a composition-specific Fe-BDC content.

### 2.4. Experimental Setup for Column Seepage Tests

#### 2.4.1. Research Objectives

This study aims to investigate the remediation performance of PET-Fe-BDC composite geotextiles for heavy metal contamination (Pb^2+^ and Cd^2+^) in flowing groundwater environments, with a particular focus on dynamic retention behavior and breakthrough modeling. First, an iron–terephthalate-containing Fe-BDC phase was synthesized in situ on the PET geotextile surface, followed by PXRD, FTIR, XPS, and SEM characterization of its crystalline features, chemical bonding environments, surface composition, and morphology. Subsequently, column seepage experiments were conducted to simulate groundwater flow, comparing the performance of plain soil systems with composite-geotextile-reinforced soil systems to evaluate their dynamic retention performance and migration behavior for Pb^2+^ and Cd^2+^. Finally, the Thomas, Yan, Clark, and Yoon–Nelson models were applied to quantitatively compare the breakthrough behavior of the unamended and composite-geotextile-amended soil systems and to obtain characteristic dynamic retention parameters.

#### 2.4.2. Experimental Apparatus

The column apparatus is shown in Figure 4 and Figure 5. The experiments were performed in acrylic glass columns with an inner diameter of 5 cm and a height of 22 cm. Sampling ports were installed along the column at 5 cm intervals to monitor the seepage process. Each column contained a 20 cm high soil bed. Two experimental configurations were established: an unamended-soil system and a PET-Fe-BDC composite-geotextile-amended soil system. Each configuration included three independently packed columns (*n* = 3), resulting in a total of six columns. All replicate columns were operated under identical influent and hydraulic conditions. Pb(II) and Cd(II) were introduced simultaneously in the same mixed influent. A 2 cm thick layer of quartz sand (1–3 mm in diameter) was placed at the top of the column to act as a buffer layer. Filter paper was placed at the column base, within the sampling ports, and atop the quartz sand layer to prevent clogging of the inlets and outlets. The unamended-soil columns served as the reference for the intrinsic retention and transport behavior of the soil, whereas the PET-Fe-BDC composite-geotextile-amended columns were used to evaluate the net remediation performance of the complete composite system under identical seepage conditions.

#### 2.4.3. Experimental Procedure

The influent consisted of a composite heavy metal solution containing 20 mg/L each of Pb^2+^ and Cd^2+^. The flow rate was precisely controlled using a peristaltic pump (F01A-STP, Kamoer Fluid Tech (Shanghai) Co., Ltd., Shanghai, China). Prior to the experiment, the four lateral sampling ports were closed. The soil columns were saturated by pumping deionized water at the target flow rate for 48 h, or until a uniform discharge was observed at the bottom outlet. Subsequently, the influent was switched to the heavy metal solution, which was pumped into the column and collected from the bottom outlet. Liquid samples were collected at fixed intervals from the sampling ports, centrifuged, and analyzed using Inductively Coupled Plasma Optical Emission Spectroscopy (ICP-OES (Optima 8000, PerkinElmer, Inc., Waltham, MA, USA)) to determine the concentrations of heavy metal ions at various column depths. At each sampling time and depth, the reported concentration was calculated as the mean of three independent column measurements. Experimental variability in the principal breakthrough curves is presented as the mean ± standard deviation (SD) of the three independent columns (*n* = 3). Breakthrough times and model parameters were derived from the corresponding replicate-averaged concentration–time curves.

The open and interconnected structure of the needle-punched PET geotextile was selected to preserve cross-plane water-flow pathways within the column. During the column experiments, the influent flow rate was maintained at 60 mL/h using a peristaltic pump throughout the 252 h operating period. Referring to ISO/TS 21268-3:2007 [38], this flow rate was selected to facilitate the time-resolved evaluation of pollutant transport. An influent concentration of 20 mg/L for both Pb^2+^ and Cd^2+^ was used to provide well-resolved concentration profiles throughout the column tests. The value of 526 mg represents the total mass of the circular PET-Fe-BDC composite specimen rather than the mass of the deposited Fe-BDC fraction. The experimental setup and test parameters are shown in Figure 4 and Figure 5 and summarized in Table 3.

## 3. Results and Discussion

### 3.1. Structural and Physicochemical Characterization

#### 3.1.1. PXRD Analysis

The PXRD pattern of the independently synthesized Fe-BDC powder exhibited reflections at 2θ = 9.10°, 10.52°, 18.82°, 21.26°, and 25.88° (Figure 6a). Several principal reflections were qualitatively consistent with ICDD PDF 33-1723 for iron terephthalate hydroxide, Fe(OH)(BDC), while the additional reflections indicated that the synthesized product had a more complex crystalline composition than the single reference phase. Together with the FTIR and XPS evidence for iron–carboxylate coordination, the PXRD results support identifying the synthesized product at the compositional level as an iron–terephthalate-containing Fe-BDC material. Because the available data do not establish a unique framework topology or quantitative phase composition, Fe-BDC is used throughout this study as a composition-based designation rather than as an assignment to a specific or phase-pure MOF structure.

Activated PET displayed its characteristic diffraction features in the 15–30° region (Figure 6b). Following in situ synthesis, PET-Fe-BDC retained the diffraction features of the PET substrate and exhibited additional low-angle reflections at 2θ = 8.44° and 9.58° (Figure 6c). These reflections were absent from activated PET and provide additional PXRD evidence for the presence of the Fe-BDC-containing material on the PET substrate.

#### 3.1.2. FTIR Analysis

The FTIR spectra of Fe-BDC powder and PET-Fe-BDC exhibited common bands associated with the iron–terephthalate structure (Figure 7). The bands at approximately 1597 and 1393 cm^−1^ were assigned to the asymmetric and symmetric stretching vibrations of coordinated carboxylate groups, respectively, while the band near 750 cm^−1^ was associated with the aromatic ring. A broad band near 3400 cm^−1^ was attributed to O–H/N–H stretching. PET-Fe-BDC additionally retained the PET-associated band at approximately 1126 cm^−1^. The coexistence of the Fe-BDC-related carboxylate bands and the PET-associated band supports the successful integration of the Fe-BDC phase with the fibrous PET substrate.

#### 3.1.3. XPS Analysis

The XPS survey spectra confirmed the presence of C, O, and Fe in both Fe-BDC powder and PET-Fe-BDC, with an additional N signal in the composite associated with the PAM-containing preparation route (Figure 8a). The high-resolution Fe 2p spectra displayed features at approximately 711.1 and 724.5 eV, corresponding to Fe 2p3/2 and Fe 2p1/2, respectively (Figure 8d). The corresponding C 1s and O 1s spectra further demonstrated carbon- and oxygen-containing chemical environments in both samples (Figure 8b,c). Together with the PXRD and FTIR results, the XPS data support the presence of an iron–terephthalate-containing phase on the PET substrate.

#### 3.1.4. SEM Analysis

SEM images showed that the independently synthesized Fe-BDC powder consisted of heterogeneous elongated and faceted crystallites spanning the submicrometre-to-micrometre scale (Figure 9a,b). In PET-Fe-BDC, numerous elongated and faceted particles were distributed along the PET fiber surfaces (Figure 9c,d), while the interconnected voids between the nonwoven fibers remained visible. This particle-decorated fiber morphology is consistent with the successful deposition of the Fe-BDC-containing phase on the PET substrate.

Based on gravimetric measurements from 3 separate preparation batches (*n* = 3), the mean apparent loading fraction of the PET-Fe-BDC composite, calculated relative to the initial dry mass of the activated PET substrate, was approximately 9.33%. However, partial mass loss of the PET substrate during the preparation process may have reduced the observed net mass gain of the final composite. Therefore, the reported value should be regarded as a conservative semiquantitative estimate rather than a precise measurement of the absolute Fe-BDC loading.

PAM was incorporated as an intermediate coating step in the preparation route. Accordingly, the apparent loading and dynamic retention results are reported at the level of the integrated PET-Fe-BDC composite system.

Several important characterization limitations should also be acknowledged. Given the partial correspondence with ICDD PDF 33-1723 and the presence of additional reflections, the PXRD results are interpreted qualitatively, and no Rietveld-based phase proportions are reported. This interpretation supports the composition-based Fe-BDC designation while avoiding assignment to a specific framework topology or phase-pure structure. The apparent gravimetric loading was not independently validated using a composition-specific quantitative method, and EDS elemental mapping, TEM, and N_2_ adsorption–desorption measurements were not available in the present study. Consequently, the spatial uniformity of Fe distribution, the nanoscale morphology and interfacial structure of the deposited phase, and the BET specific surface area, pore volume, and pore-size distribution cannot be quantitatively established from the current data. The SEM and XPS results support the deposition and surface chemical composition of the Fe-BDC-containing phase, but they should not be regarded as substitutes for these measurements. Future studies should further optimize the synthesis toward a better-defined iron–terephthalate phase and incorporate quantitative phase analysis, independent loading quantification, EDS mapping, TEM, and BET analysis to establish more detailed relationships among phase composition, material loading, pore structure, and dynamic retention performance.

### 3.2. Dynamic Retention and Migration Behavior

In a continuous column seepage process, a breakthrough curve illustrates the time-dependent variation in heavy metal ion concentration in a solution as it permeates through a fixed thickness of a soil layer. It is widely utilized to characterize the migration behavior of heavy metal ions within a specific medium. Figure 10a,b present the breakthrough curves of Pb^2+^ and Cd^2+^ in the plain soil system, whereas Figure 10c,d depict the corresponding curves for the composite geotextile–soil system. The variables c_0_ and c_t_ denote the initial concentration of the heavy metal ions (mg/L) and the effluent concentration at a given sampling time (mg/L), respectively.

#### 3.2.1. Breakthrough Behavior

The column comparison was conducted at the level of the integrated PET-Fe-BDC composite-geotextile-amended soil system. Within this material configuration, PET provides a continuous and permeable supporting scaffold, PAM was included as an intermediate coating step before Fe^3+^ exposure, and Fe-BDC constitutes the deposited iron–terephthalate-containing reactive phase. The assignment of the integrated PET-Fe-BDC composite is supported by complementary characterization evidence, including the PXRD comparison between activated PET and the final composite, the N 1s and Fe 2p signals detected by XPS, the characteristic iron–terephthalate bands identified by FTIR, and the SEM observation of Fe-BDC-containing particles distributed along the PET fibers. Accordingly, the following breakthrough results represent the net performance of the complete PET-Fe-BDC composite-geotextile-amended soil system relative to the unamended-soil system.

As illustrated in Figure 10, the breakthrough curves of Pb^2+^ and Cd^2+^ in the plain soil system and the PET-Fe-BDC composite geotextile–soil system exhibit clear differences in morphology and transport characteristics.

In the plain soil system (Figure 10a,b), the breakthrough curves for both ions displayed typical S-shaped characteristics. As the bed depth increased (from 5 to 20 cm), both the breakthrough and saturation points were markedly delayed. Taking Pb^2+^ (Figure 10a) as an example, the rapid visually observed concentration-rise times at depths of 5, 10, 15, and 20 cm were 0, 720, 2160, and 7920 min, respectively, with corresponding apparent plateau times of 3600, 6480, 9360, and 13,680 min. A similar rightward shift with increasing depth was observed for Cd^2+^ (Figure 10b), where the visually observed concentration-rise times at 5, 10, 15, and 20 cm were 0, 720, 2160, and 5040 min, and the apparent plateau times were 3600, 5040, 7920, and 9360 min, respectively. These visually identified transition times were used only to describe the changes in breakthrough-curve morphology. For the unamended-soil columns, the formal breakthrough time (*t*_b_) and saturation time (*t*_e_), defined at *c_t_*/*c*_0_ = 0.10 and 0.95, respectively, were calculated by interpolation. This phenomenon is attributed to the progressive downward migration of the mass transfer zone within the low-liquid-limit clay layer during the initial seepage stage. However, due to the limited adsorption capacity of the plain soil, the effluent concentrations at all depths eventually approached the influent concentration (*c_t_*/*c*_0_ > 0.8) after rapid breakthrough, indicating substantial exhaustion of the retention sites, although the formal saturation criterion of *c_t_*/*c*_0_ = 0.95 was not necessarily reached.

Compared with the unamended-soil system, the PET-Fe-BDC composite-geotextile-amended soil system exhibited substantially delayed heavy-metal migration and a pronounced concentration-truncation effect (Figure 10c,d). In the shallow-to-middle layers (5–15 cm), the effluent concentrations increased but never reached complete breakthrough. For Pb^2+^ (Figure 10c), the visually observed concentration-rise times at 5, 10, and 15 cm were substantially delayed to 2160, 3600, and 5040 min, reaching quasi-equilibrium states at approximately 6480, 10,800, and 12,240 min, respectively. At steady state, the peak effluent concentrations at 5 and 10 cm were reduced by approximately 70% (*c_t_*/*c*_0_ maintained between 0.25 and 0.28), while the reduction rate at 15 cm reached approximately 80% (*c_t_*/*c*_0_ decreased to about 0.2). In the Cd^2+^ system (Figure 10d), the visually observed concentration-rise times at 5, 10, and 15 cm were delayed to 720, 2160, and 3600 min, reaching quasi-equilibrium at 6480, 6480, and 7920 min, respectively. The peak effluent concentrations at 5 and 10 cm were reduced by approximately 60% (*c_t_*/*c*_0_ = 0.36–0.42), with a 65% reduction at 15 cm (*c_t_*/*c*_0_ ≈ 0.35). Regarding deep-layer interception, the composite system showed superior barrier performance: while the plain soil column was ultimately breached at a depth of 20 cm, the composite system achieved an interception reduction rate of over 90% at the same depth (*c_t_*/*c*_0_ remained below 0.1 throughout), demonstrating near-complete containment of the ions.

Regarding dynamic temporal characteristics, the composite system not only markedly delayed the breakthrough initiation but also extended the time interval required to reach quasi-equilibrium from initial breakthrough. Data show that the time intervals for Pb^2+^ in the plain soil system at 5, 10, and 15 cm were 3600, 5760, and 7200 min, respectively, whereas in the composite system, these intervals were extended to 4320, 7200, and 7200 min. The Cd^2+^ system exhibited a similar trend: the intervals for plain soil at 5, 10, and 15 cm were 3600, 2880, and 2880 min, which were extended to 5760, 4320, and 4320 min in the composite system. The extension of these intervals and the reduction in the breakthrough curve slope indicate that the incorporation of the composite material increased the apparent internal mass-transfer resistance. This increased residence time and enhanced contact between heavy metal ions and the medium, thereby producing sustained breakthrough retardation throughout the 252 h continuous-loading period.

Further comparison of the two ions in the composite system (Figure 10c,d) revealed ion-dependent retention. The quasi-equilibrium concentration plateau for Pb^2+^ (0.25–0.28) was consistently lower than that for Cd^2+^ (0.36–0.42) at the same depth, corresponding to an approximately 15% greater concentration reduction for Pb^2+^. This behavior is consistent with the generally stronger affinity of Pb(II) for oxygen-containing functional groups reported in previous studies [39,40,41]. Overall, the PET-Fe-BDC composite-geotextile-amended soil system substantially delayed the breakthrough and downward migration of both ions, with stronger retardation observed for Pb(II).

#### 3.2.2. Depth- and Time-Dependent Migration

The migration profiles of Pb^2+^ and Cd^2+^ within the plain soil and composite systems reveal distinct evolution patterns throughout the experimental duration, as shown in Figure 11.

During the initial stage (12 h, Figure 11a), initial differences in migration behavior were observed. In the plain soil column, both ions exhibited a tendency to penetrate toward deeper layers. In contrast, the composite-geotextile-amended system exhibited pronounced retention in the shallow layers, resulting in lower Pb(II) and Cd(II) concentrations and delayed downward migration compared with the unamended soil system.

At a depth of 5–10 cm, Pb^2+^ in the plain soil system had not yet broken through, whereas Cd^2+^ showed slight downward migration. As depth increased to 10–20 cm, concentration gradients in both systems remained relatively low, suggesting that the pollution front had not yet migrated appreciably into the deeper sections of the columns.

As the operation time extended to 84 h (Figure 11b), the retention front gradually moved downward. In the 0–5 cm zone, both systems experienced ion breakthrough, albeit to different degrees: the concentration in the plain soil column approached the influent level, indicating shallow-layer saturation, whereas the composite system maintained substantially lower concentrations, demonstrating persistent retention capacity. At a depth of 5–10 cm, Cd^2+^ in the plain soil column was nearly fully saturated (reaching approximately 80% of the influent concentration at 10 cm), and Pb^2+^ also exhibited extensive breakthrough, with Cd^2+^ demonstrating higher mobility than Pb^2+^. Conversely, the composite system effectively delayed the breakthrough process; notably, the Pb^2+^ concentration at 10 cm was only approximately 1% of the influent value. In the deeper 10–15 cm region, Pb^2+^ and Cd^2+^ concentrations in the plain soil reached 15% and 35% of the influent, respectively, compared to only 5% and 10% in the composite system. By 20 cm, neither system had achieved full breakthrough.

By 156 h (Figure 11c), the concentrations of Cd(II) throughout the 0–20 cm soil profile and those of Pb(II) within the 0–15 cm region of the unamended soil column had approached the influent concentration, indicating extensive breakthrough. In contrast, the composite-geotextile-amended system maintained substantially lower ion concentrations and a pronounced concentration gradient along the soil profile.

At the final experimental stage of 252 h (Figure 11d), the ion concentrations in the unamended soil column remained close to the influent concentration throughout the 0–20 cm depth. The composite-geotextile-amended system continued to exhibit lower concentrations and delayed downward migration of both ions. These results demonstrate that incorporation of the PET-Fe-BDC geotextile substantially prolonged the contaminant-retardation performance of the soil column under continuous seepage conditions.

Continuous effluent collection and depth-resolved sampling were maintained throughout the 252 h operating period under the imposed constant-flow condition. The complete time-series data therefore demonstrate sustained operation and contaminant-retardation performance of the PET-Fe-BDC-containing column under the tested seepage conditions.

#### 3.2.3. Quantitative Dynamic Retention

Dynamic retention was quantitatively evaluated using the formal breakthrough time (tb), cumulative retained mass (qtot), total influent mass (mtot), and cumulative removal efficiency (Y), following established approaches for continuous-flow column analysis [42,43,44]. Breakthrough was defined at *c_t_*/*c*_0_ = 0.10, while *t_e_*′ represented the transition point into the slow-retention stage of the composite-system breakthrough curves. The detailed calculation equations and parameter definitions are provided in Supplementary Note S1. The resulting dynamic retention parameters are summarized in Table 4.

Figure 12 illustrates the variation curves of breakthrough time with column height during the seepage of Pb^2+^ and Cd^2+^ in both the plain soil and composite geotextile–soil systems. As depicted, the breakthrough times for both Pb^2+^ and Cd^2+^ increased with increasing column height. Under identical conditions, the breakthrough time for Pb^2+^ was consistently longer, indicating that Cd^2+^ exhibited a faster migration rate in both the plain soil and the composite systems. In the 0–5 cm soil layer, the formal breakthrough times increased from 621.72 to 4294.51 min for Pb^2+^ and from 337.75 to 2146.75 min for Cd^2+^ after the incorporation of the composite geotextile. Thus, the breakthrough times increased to 6.91 and 6.36 times those of the unamended soil system, respectively. Further analysis regarding the effect of column height on the extension ratio of breakthrough time (Figure 13) demonstrated that as the column height increased, the impact of the composite geotextile layer on the breakthrough times of Pb^2+^ and Cd^2+^ gradually diminished. This indicates that the retardation effect of the PET-Fe-BDC composite-geotextile-amended soil system was concentrated primarily in the shallow soil layers.

Comparison of the cumulative retained masses (*q*_tot_) of Pb^2+^ and Cd^2+^ during the seepage process showed stronger retention of Pb^2+^ by the low-liquid-limit clay system. This ion-dependent behavior is consistent with the intrinsic differences in the physicochemical properties of the two metals. Studies have indicated [45] that during migration in soil, the adsorption of Pb^2+^ primarily occurs at variable charge sites within the binary system, whereas the adsorption of Cd^2+^ mainly takes place at permanent charge sites. Consequently, oxygen-containing functional groups on the adsorbent surface are more prone to react with Pb^2+^. Research has also demonstrated that aromatic compounds can act as π-electron donors [46], forming complexes with Cd^2+^ to enhance its adsorption; however, the presence of Pb^2+^ inhibits such complexation reactions. Furthermore, the efficacy of a solid adsorbent in extracting solutes from water is highly dependent on the hydrochemical properties of the solutes—specifically, the affinity between the solute and the solvent, chemically defined as solubility [47]. Before heavy metal ions can be adsorbed onto the adsorbent surface, they must first desorb from the aqueous phase. Therefore, compounds with stronger affinity to the aqueous solution and higher solubility are more difficult to desorb. Comparing the solubilities of Pb(NO_3_)_2_ and Cd(NO_3_)_2_ at 20 °C, which are 54 g/100 mL H_2_O and 136 g/100 mL H_2_O, respectively, it is evident that Pb^2+^ desorbs more readily from the solution. The lower hydrolysis capability of Cd^2+^ prevents it from effectively competing for adsorption sites on variable charge surfaces, making its adsorption more limited. Macroscopically, this is reflected in the stronger retention of Pb^2+^ than Cd^2+^ by the soil system. Because the composite system did not reach complete saturation, the inflection time *t*_e_′ was selected as the evaluation endpoint. At the respective *t*_e_′ values (7200 min for Pb^2+^ and 6480 min for Cd^2+^), the cumulative amounts of Pb^2+^ and Cd^2+^ retained by the composite geotextile–soil system in the 0–5 cm zone were 130.01 and 104.07 mg, respectively. The corresponding cumulative removal efficiencies were 90.29% and 80.30%, demonstrating effective dynamic retention of both heavy metal ions under continuous seepage conditions.

Because effluent Fe concentrations were not monitored, the possible contributions of Fe release and Fe-associated precipitation to the overall immobilization of Pb(II) and Cd(II) could not be independently quantified. This limitation does not alter the directly measured breakthrough delay, cumulative retained mass, and removal efficiency, but it restricts attribution of the observed retention to a single mechanism. Accordingly, the results are interpreted in terms of the system-level dynamic retention of the complete soil–composite system.

Furthermore, the PET-Fe-BDC composite was not structurally or chemically characterized after the column experiments. Consequently, the post-operation stability of the deposited Fe-BDC-containing phase and the extent of any structural or chemical changes induced during the breakthrough tests remain to be determined. Future work should therefore include post-use characterization of the composite by PXRD, FTIR, XPS, and SEM, supplemented by EDS elemental mapping, effluent Fe analysis, mass-balance assessment, hydraulic-property monitoring, and regeneration experiments, to evaluate its structural evolution and long-term operational stability.

### 3.3. Comparative Evaluation of Breakthrough Models

#### 3.3.1. Thomas Model Fitting Analysis

The Thomas model was applied to evaluate the breakthrough behavior of Pb(II) and Cd(II) at different sampling depths [48,49]. Because the retardation effect of the PET-Fe-BDC composite-geotextile-amended soil system was most pronounced in the shallow region, the representative fitting results at the 0–5 cm sampling depth are presented in Figure 14. The complete model formulation, full-depth fitting results, and detailed fitting parameters are provided in Supplementary Note S2, Figure S5, and Table S1.

At the 0–5 cm sampling depth, the Thomas model produced R^2^ values of 0.9995 and 0.9983 for Pb(II) and Cd(II), respectively, in the unamended soil system. In the PET-Fe-BDC-amended system, the corresponding R^2^ values were 0.8667 and 0.9999. Thus, the unamended-soil data and the composite-system Cd(II) data followed the transformed Thomas relationship closely over the measured interval, whereas the lower R^2^ value for Pb(II) in the composite system indicated a clear deviation from ideal Thomas-model behavior.

The experimental *q*_tot_ values in Table 4 and the Thomas-fitted *q_M_* values in Supplementary Table S1 represent different quantities. The former is the cumulative retained mass obtained by numerical integration up to the selected evaluation endpoint, whereas the latter is a model-derived parameter obtained by fitting and extrapolating the breakthrough curve. For Pb(II) in the composite system at 0–5 cm, the experimental *q*_tot_ was 130.01 mg, while the Thomas-fitted *q_M_* was 239.30 mg. This difference, together with the lower R^2^ value of 0.8667, reflects the incomplete and non-ideal breakthrough profile of Pb(II), which weakly constrained the extrapolated Thomas parameter. In contrast, the Cd(II) curve more closely followed the Thomas relationship over the measured interval, producing a fitted *q_M_* of 103.26 mg, an experimental *q*_tot_ of 104.07 mg, and an R^2^ value of 0.9999. Nevertheless, because complete saturation was not experimentally reached in the composite system, the fitted *q_M_* values are interpreted as model-derived parameters rather than experimentally verified retention capacities.

Figure 15 compares the endpoint-integrated experimental *q*_tot_ values with the Thomas-fitted *q_M_* parameters across the investigated sampling depths. Close agreement was obtained for several curves, whereas larger deviations occurred for the non-saturated composite-system curves, particularly for Pb(II). The comparison is therefore used to evaluate the descriptive performance and extrapolation behavior of the Thomas model rather than to treat *q_M_* as an experimentally measured capacity.

#### 3.3.2. Yan Model Analysis

The Yan model was applied to the representative breakthrough data obtained at the 0–5 cm sampling depth [48,50]. The complete model formulation, linearized equation, and detailed fitting parameters are provided in Supplementary Note S2 and Table S2.

As shown in Figure 16, the Yan model provided consistently strong descriptions of the experimental data. The R2 values were 0.9466 and 0.9727 for Pb(II) and Cd(II), respectively, in the unamended soil system, and 0.8738 and 0.8933 in the PET-Fe-BDC-amended system. These results indicate that the Yan model effectively captured the principal breakthrough characteristics of both metal ions.

The *q_y_m* term derived from the Yan equation provides an empirical description of the overall retention response of the soil–composite column. Because the composite-system curves did not reach complete saturation during the 252 h experiment, the Yan analysis was used primarily to compare the observed breakthrough characteristics of Pb(II) and Cd(II). Experimental retention was evaluated independently from the concentration–time data using the cumulative retained mass *q*_tot_ and removal efficiency (*Y*).

#### 3.3.3. Clark Model Analysis

The Clark model was applied to evaluate the breakthrough behavior of Pb(II) and Cd(II) at the 0–5 cm sampling depth [51]. The corresponding Clark-model fitting results are presented in Figure 17. The complete model formulation, fitting curves, and detailed parameters are provided in Supplementary Note S2 and Table S3. The model produced R^2^ values ranging from 0.6086 to 0.8093 and captured the general breakthrough trends of both metal ions. Its inclusion provides a complementary basis for comparing the applicability of different breakthrough models to the heterogeneous soil–composite system.

#### 3.3.4. Yoon–Nelson Model Analysis

The Yoon–Nelson model was also applied to the 0–5 cm breakthrough data to evaluate its representation of the characteristic breakthrough time [52]. The corresponding Yoon–Nelson-model fitting results are presented in Figure 18. The complete model formulation, fitting curves, and detailed parameters are provided in Supplementary Note S2, and Table S4. The resulting R^2^ values ranged from 0.5774 to 0.7890. These results provide an additional reference for evaluating the applicability of commonly used column models to the present binary and heterogeneous flow system.

## 4. Conclusions

In this study, the dynamic retention and migration behavior of Pb(II) and Cd(II) in an integrated PET-Fe-BDC composite-geotextile-amended soil system was systematically evaluated through column seepage experiments. The composite geotextile contained an iron–terephthalate phase prepared via an in situ solvothermal synthesis route. The main conclusions are as follows:

(1) A PET-Fe-BDC composite geotextile was prepared through sequential PET activation, PAM pretreatment, Fe^3+^ preloading, and in situ solvothermal synthesis. The PXRD pattern exhibited several reflections qualitatively consistent with ICDD PDF 33-1723, supporting the iron–terephthalate-related crystalline character of the synthesized material. FTIR, XPS, and SEM results further demonstrated the presence and deposition of the Fe-BDC-containing phase on the PET fibers while preserving the interconnected fibrous structure of the geotextile.
(2) Compared with the unamended-soil system, the PET-Fe-BDC composite-geotextile-amended soil system exhibited stronger dynamic retention of Pb^2+^ and Cd^2+^ under seepage conditions. In the 0–5 cm soil layer, the breakthrough times of Pb^2+^ and Cd^2+^ increased to approximately 6.9 and 6.4 times those in the unamended-soil system, respectively, while the endpoint-based cumulative removal efficiencies exceeded 80%. These results demonstrate the substantial retardation of downward heavy-metal migration in shallow soil layers.
(3) At the selected endpoint *t*_e_′, the composite geotextile–soil system retained 130.01 mg of Pb^2+^ and 104.07 mg of Cd^2+^, with cumulative removal efficiencies of 90.29% and 80.30%, respectively, demonstrating effective dynamic retention of both heavy metal ions.
(4) Model performance varied by ion and system. The Yan model provided the most consistent empirical description across the tested curves. The Thomas model closely described the unamended-soil curves and the composite-system Cd(II) curve, whereas its fit for composite-system Pb(II) was less satisfactory and was retained for descriptive comparison. The Clark and Yoon–Nelson models showed lower fitting performance. Model-derived parameters were interpreted on a curve-specific basis, while cumulative retained mass and removal efficiency provided direct measures of dynamic retention performance.
(5) During the 252 h column experiment, the PET-Fe-BDC composite-geotextile-amended soil system maintained lower Pb(II) and Cd(II) concentrations and delayed breakthrough relative to the unamended soil system. These results demonstrate sustained dynamic retention under the tested seepage conditions and support the potential application of the composite geotextile as a reactive component in permeable reactive barriers. However, because post-use characterization and effluent Fe analysis were not performed, the post-operation stability of the composite and any structural or chemical changes occurring during the breakthrough experiments remain to be evaluated in future studies.

## Figures and Tables

**Figure 1:**
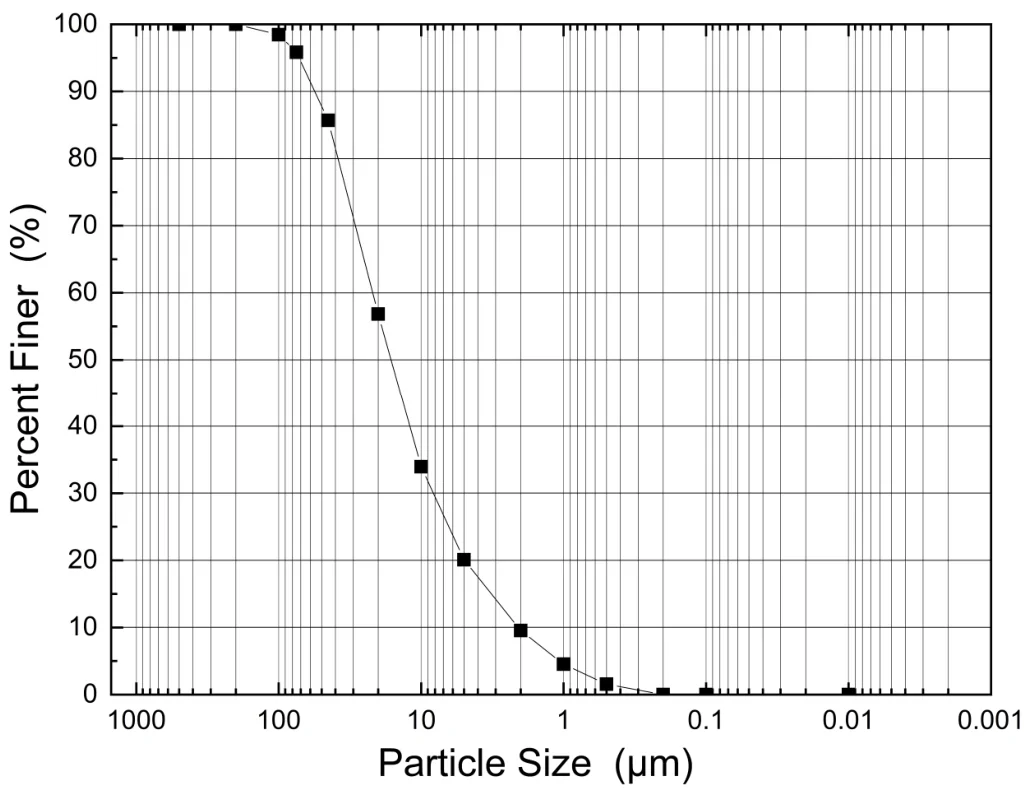
Particle size distribution curve of the test soil.

**Figure 2:**
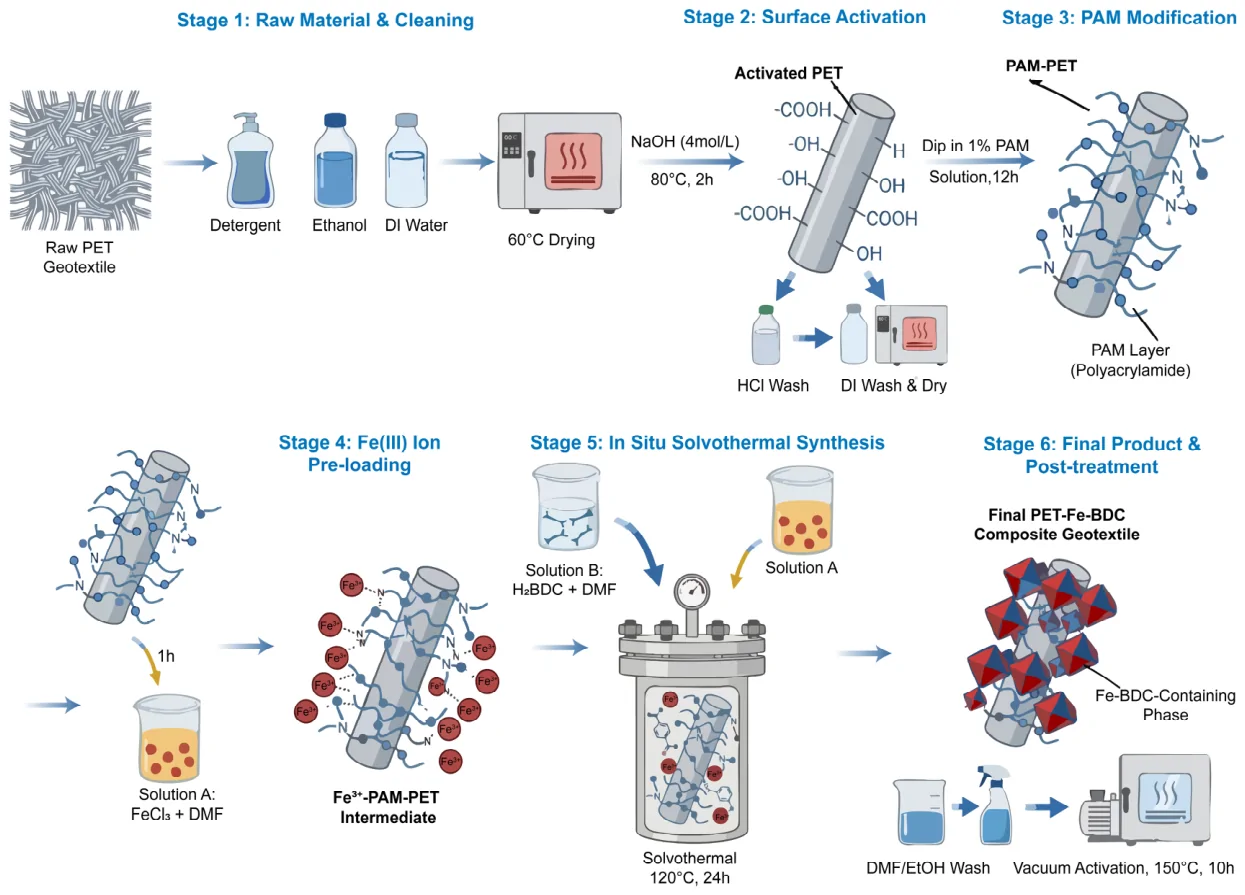
Schematic illustration of the in situ synthesis process for the PET-Fe-BDC composite.

**Figure 3:**
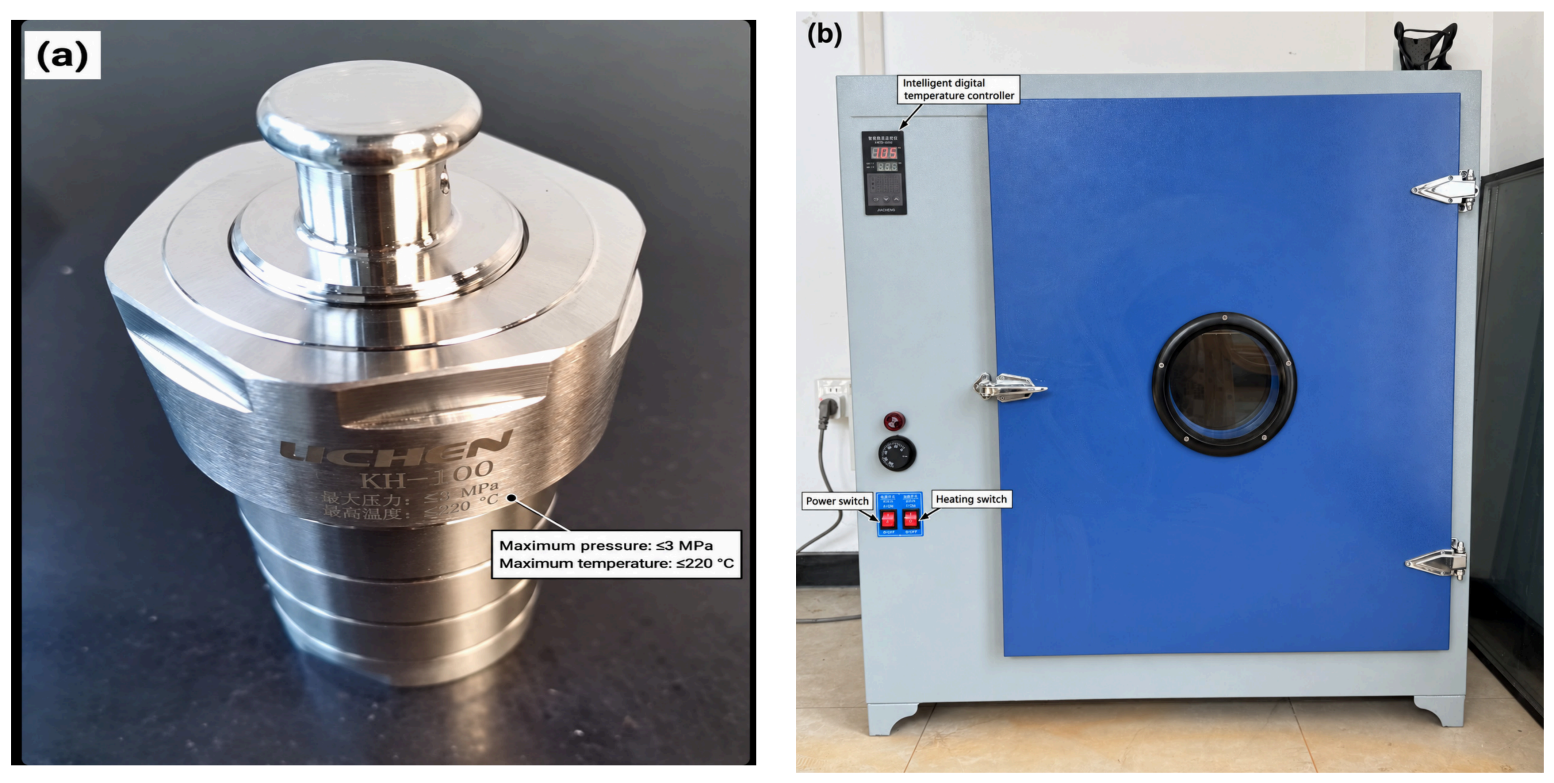
Equipment used for the preparation of PET-Fe-BDC composites: (**a**) autoclave and (**b**) forced-air drying oven.

**Figure 4:**
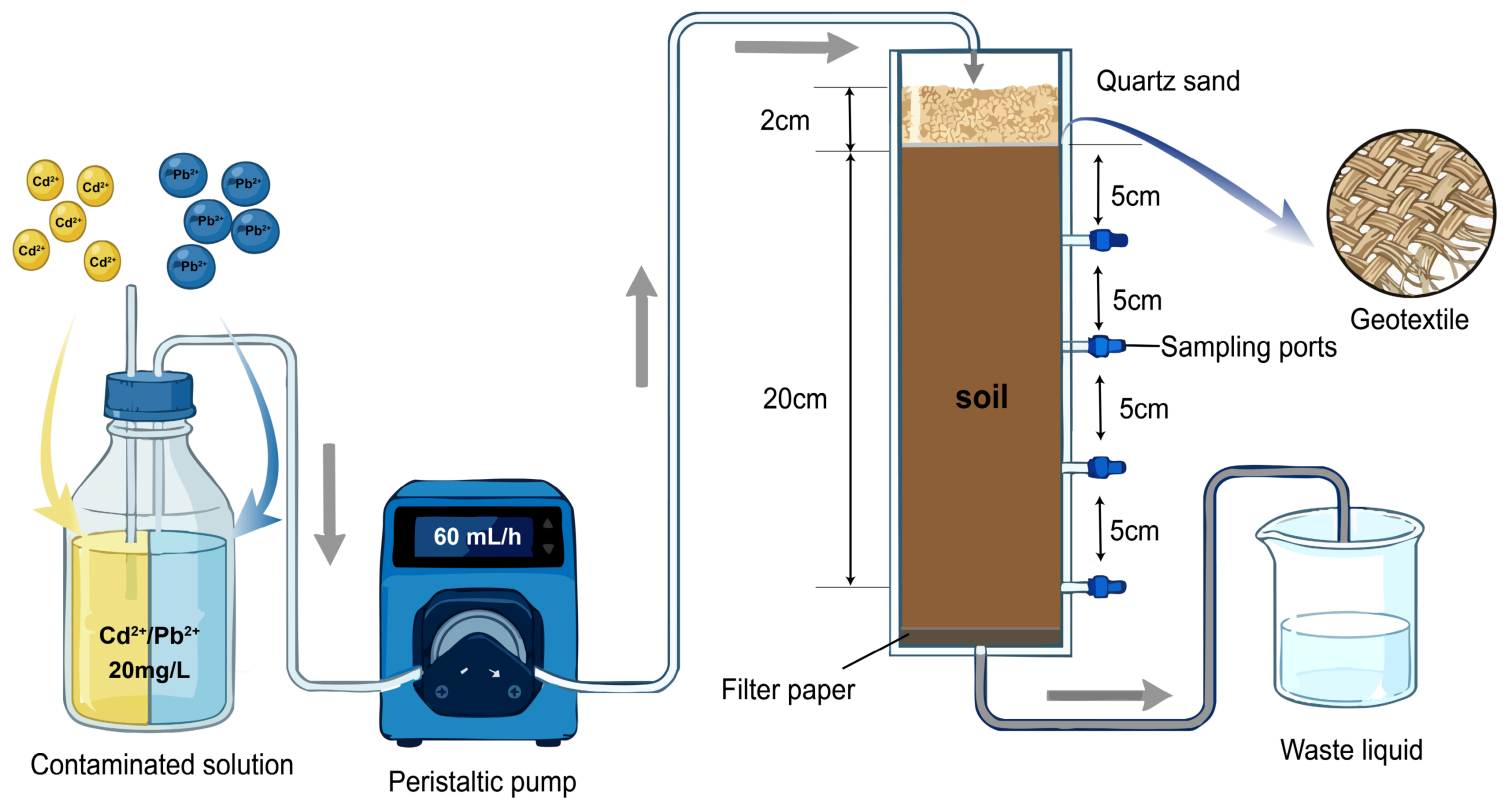
Schematic diagram of the soil column experimental setup.

**Figure 5:**
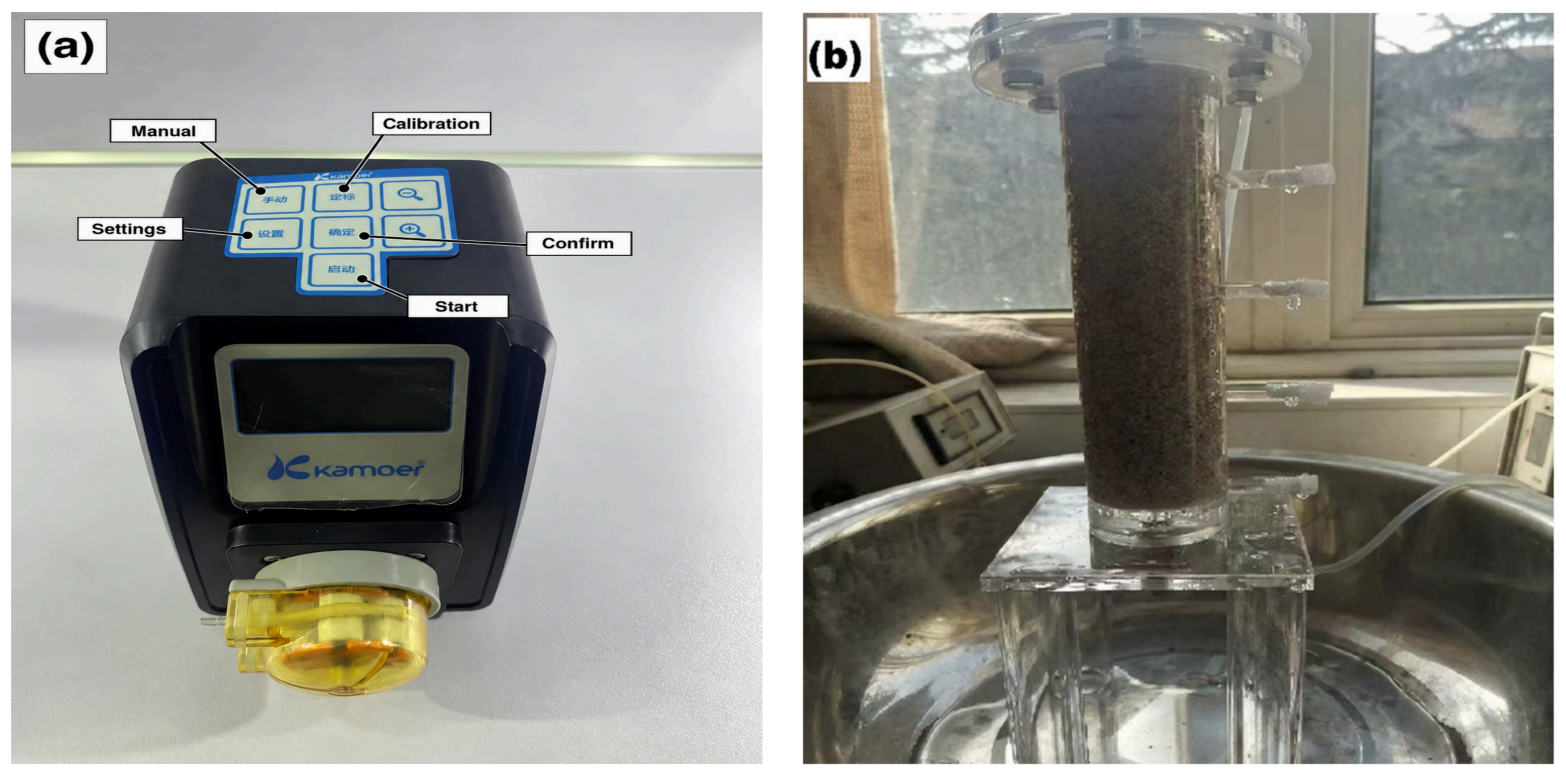
Experimental setup for the soil column tests: (**a**) micro peristaltic pump and (**b**) soil column setup.

**Figure 6:**
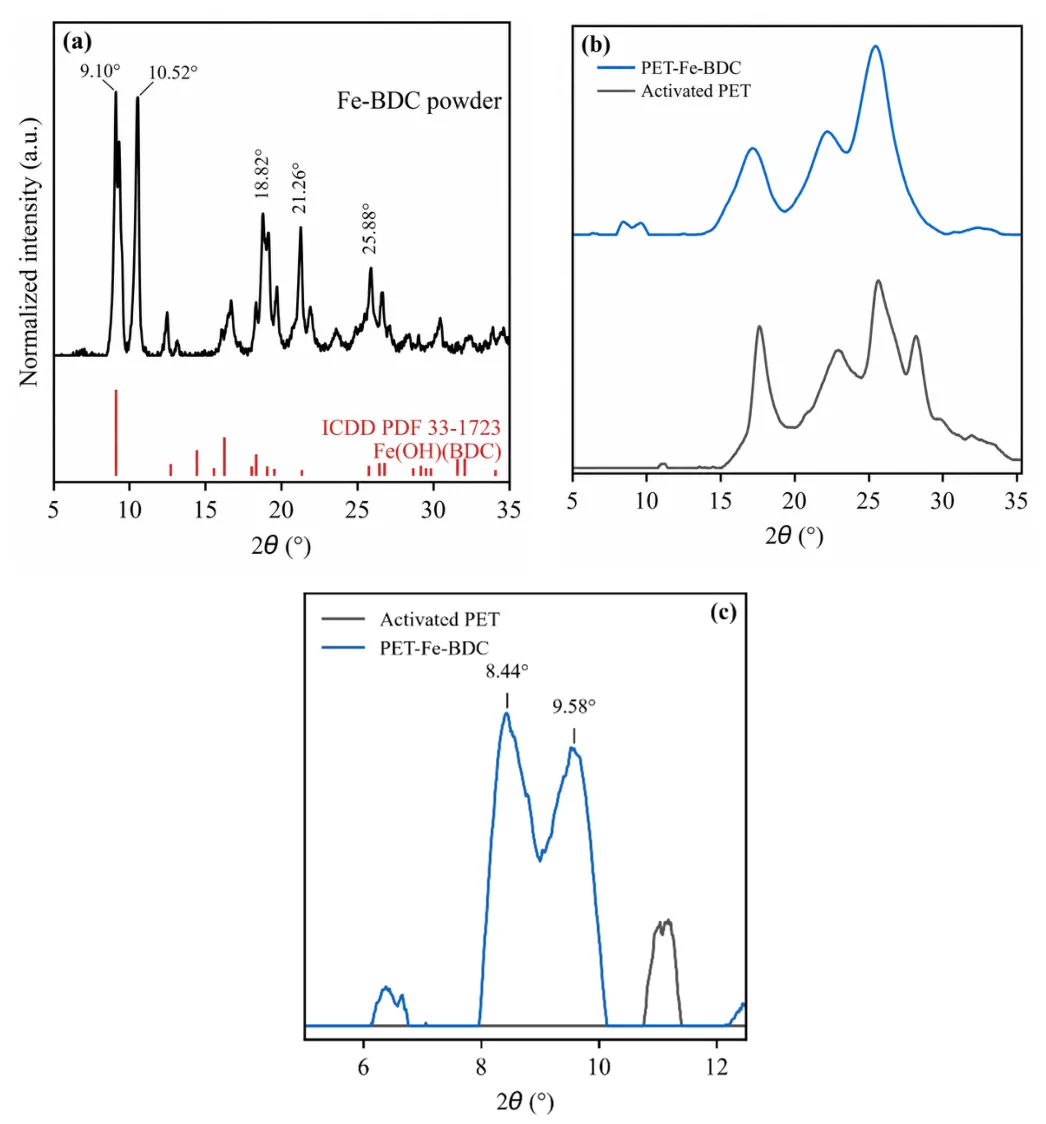
PXRD characterization of Fe-BDC and PET-Fe-BDC: (**a**) independently synthesized Fe-BDC powder qualitatively compared with ICDD PDF 33-1723 for Fe(OH)(BDC); (**b**) PXRD patterns of activated PET and PET-Fe-BDC; and (**c**) enlarged low-angle region showing the additional reflections at 2θ = 8.44° and 9.58°.

**Figure 7:**
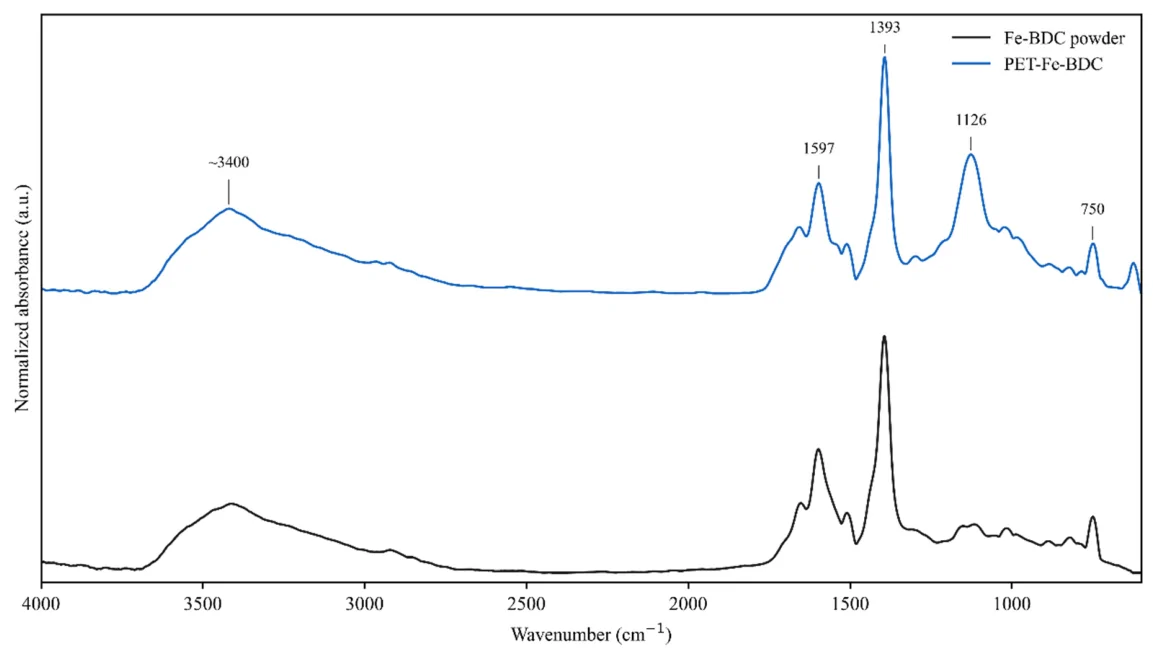
FTIR spectra of independently synthesized Fe-BDC powder and PET-Fe-BDC composite geotextile.

**Figure 8:**
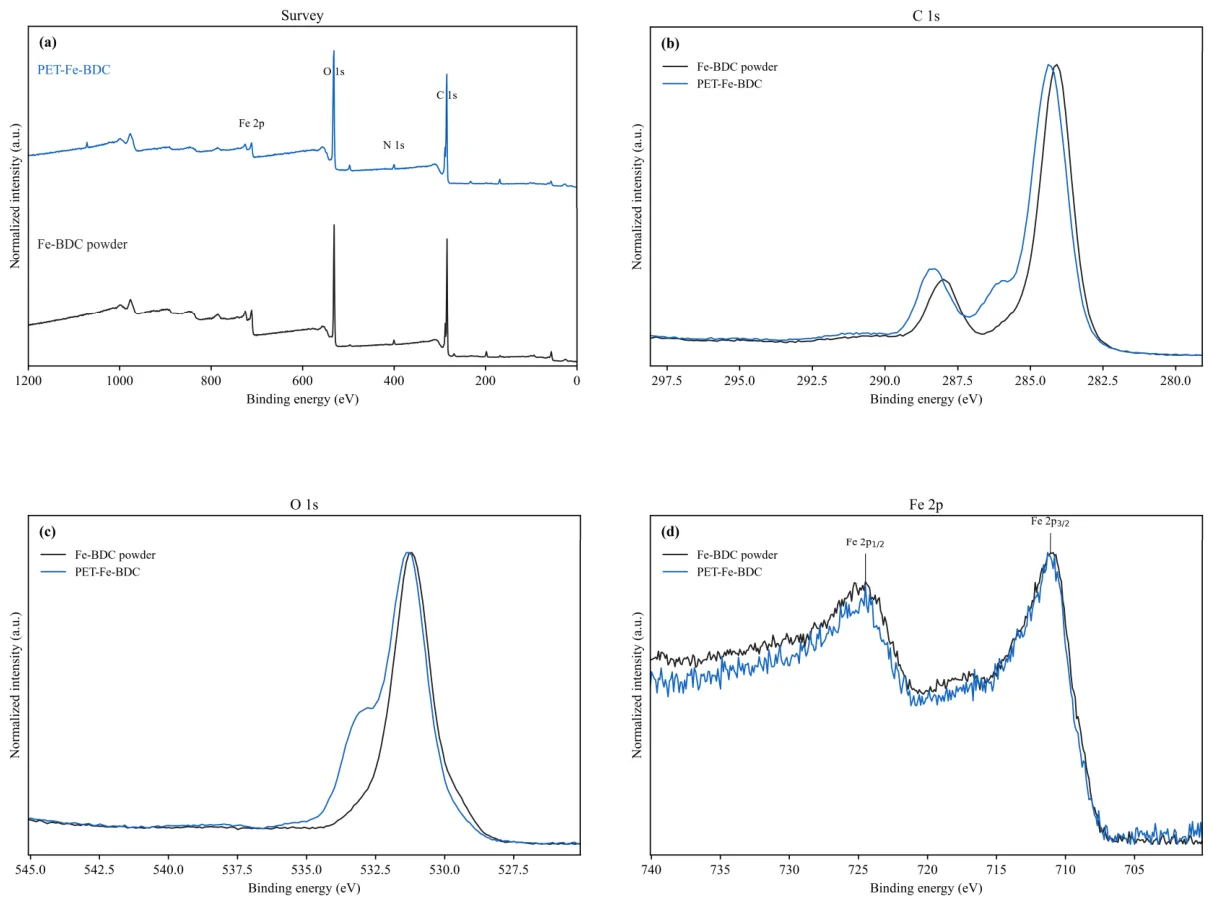
XPS spectra of Fe-BDC powder and PET-Fe-BDC: (**a**) survey spectra; (**b**) C 1s spectra; (**c**) O 1s spectra; and (**d**) Fe 2p spectra.

**Figure 9:**
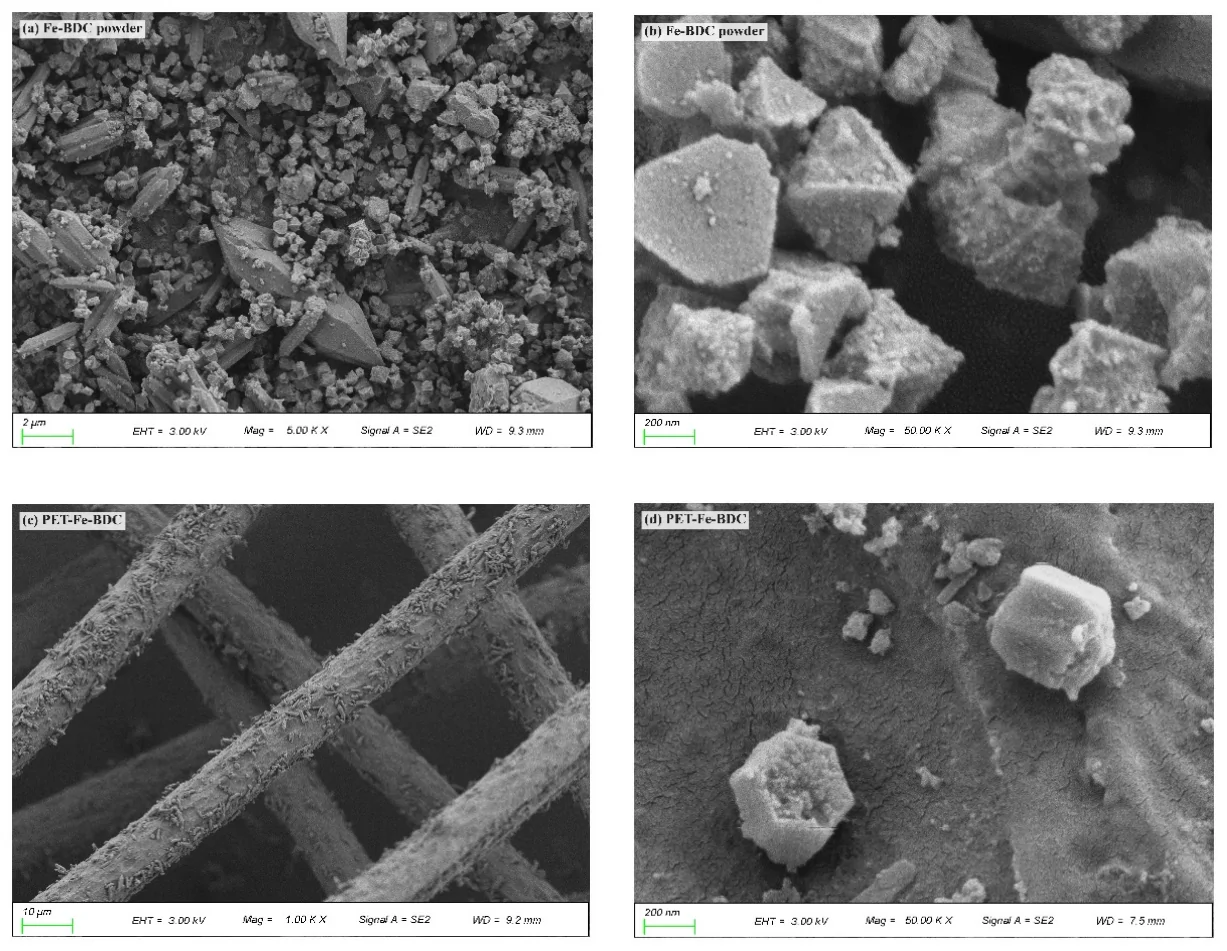
SEM micrographs of (**a**,**b**) independently synthesized Fe-BDC powder and (**c**,**d**) PET-Fe-BDC composite geotextile at complementary magnifications.

**Figure 10:**
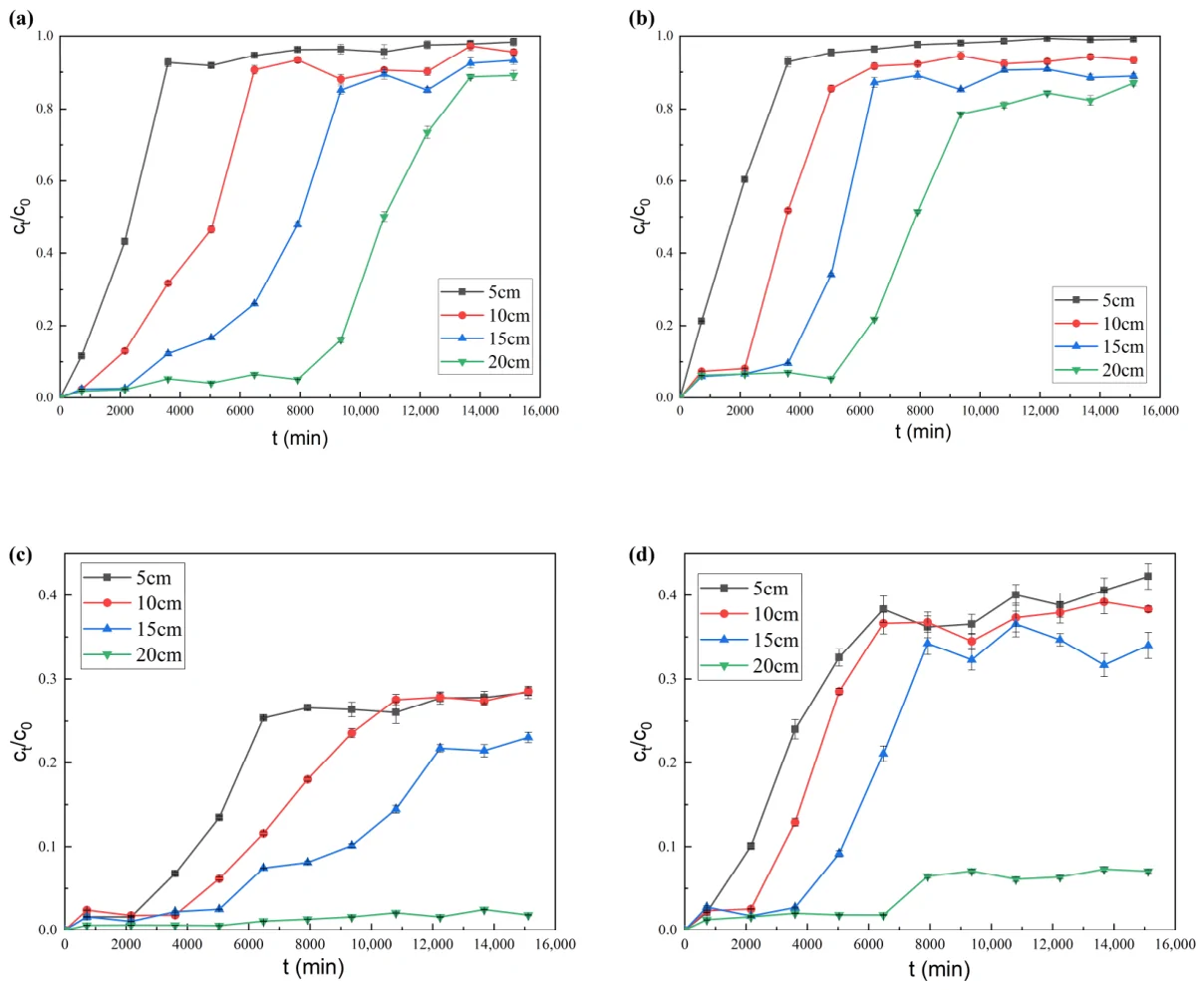
Breakthrough curves of Pb^2+^ and Cd^2+^ in unamended soil and composite-geotextile-amended soil: (**a**) Pb^2+^ in unamended soil; (**b**) Cd^2+^ in unamended soil; (**c**) Pb^2+^ in composite-geotextile-amended soil; and (**d**) Cd^2+^ in composite-geotextile-amended soil. Data are presented as mean ± standard deviation (*n* = 3 independent columns).

**Figure 11:**
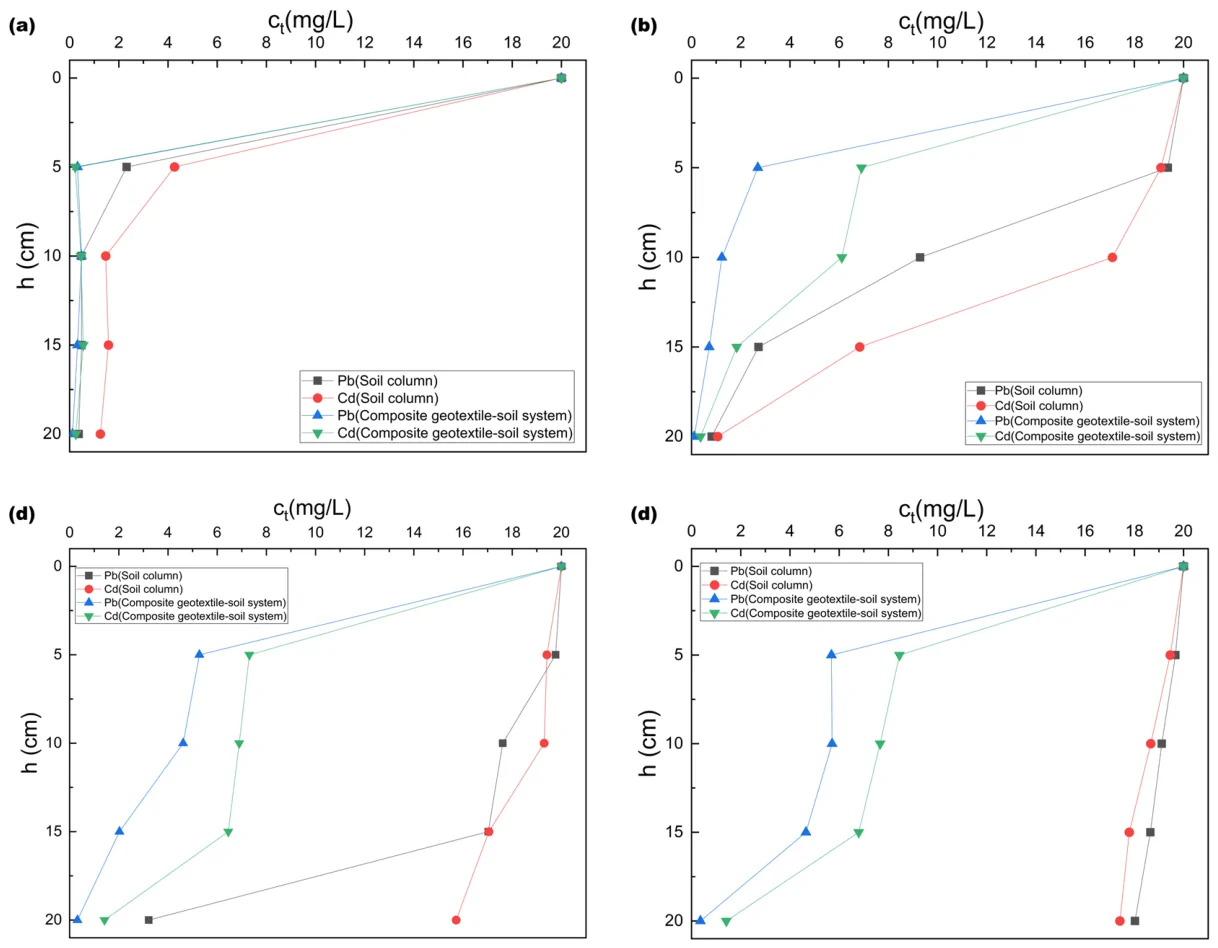
Migration profiles of Pb^2+^ and Cd^2+^ in unamended soil and composite geotextile-amended soil at different times: (**a**) 12 h; (**b**) 84 h; (**c**) 156 h; and (**d**) 252 h.

**Figure 12:**
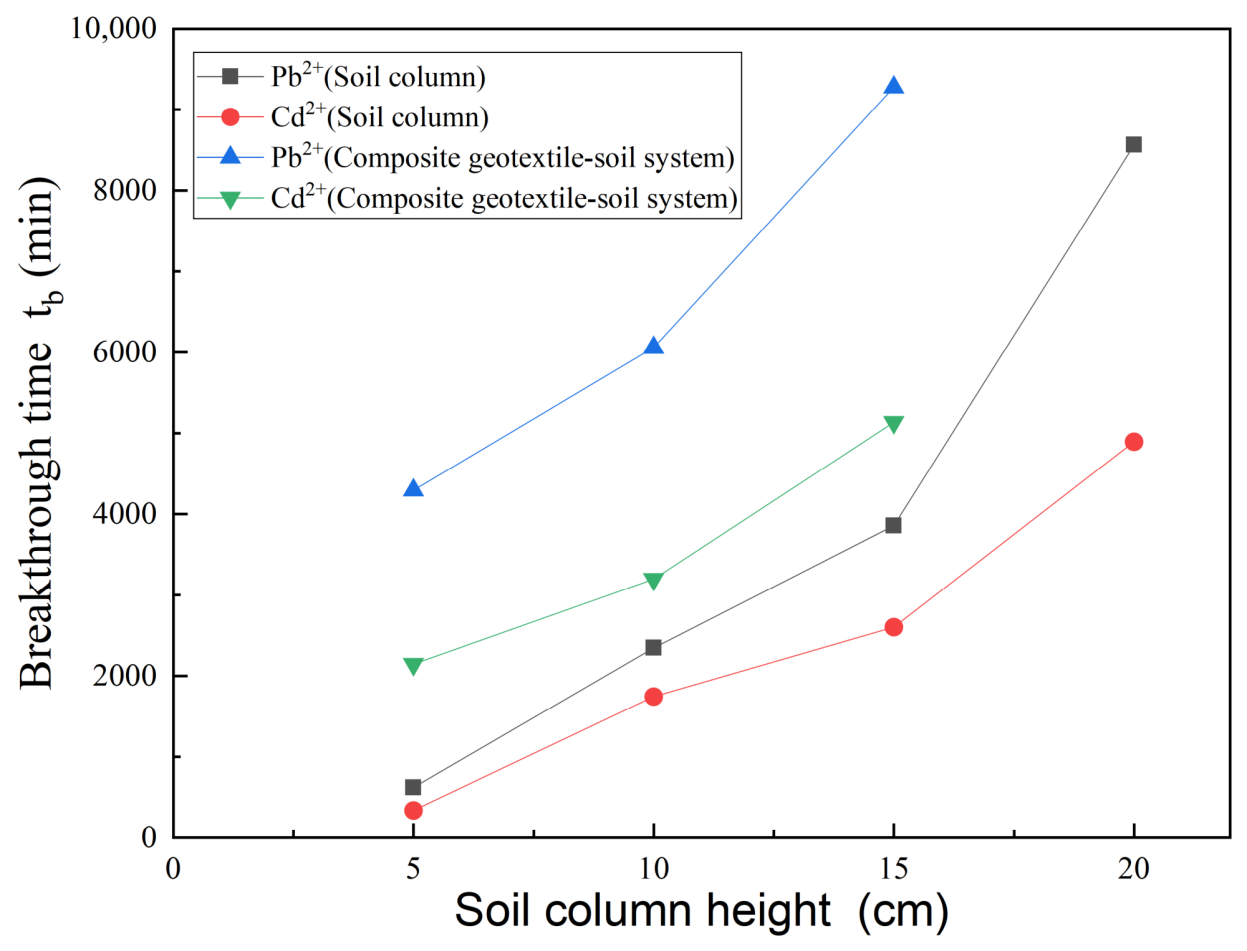
Variation in breakthrough time with soil column height.

**Figure 13:**
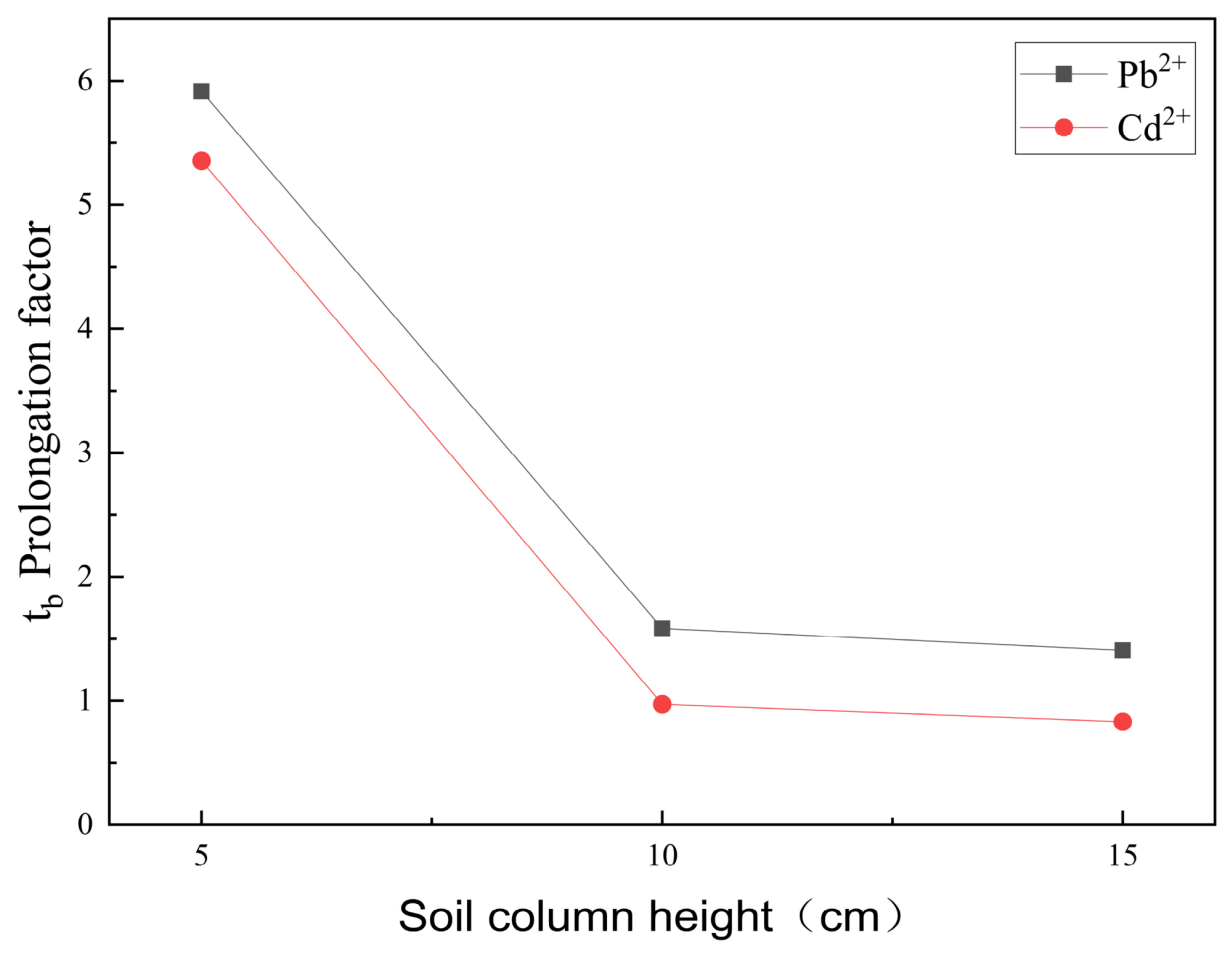
Effect of soil column height on the fold extension of breakthrough time.

**Figure 14:**
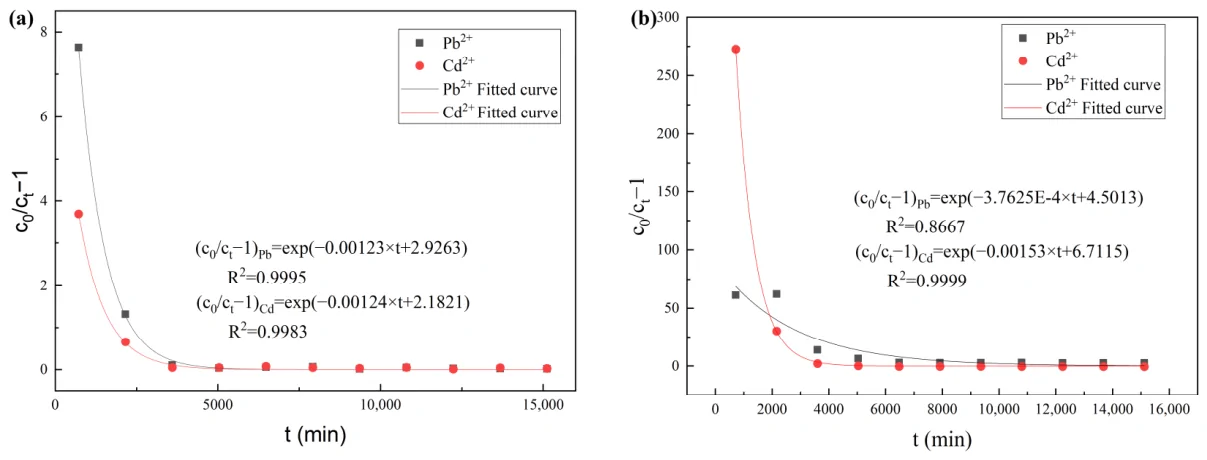
Thomas-model fitting of Pb(II) and Cd(II) breakthrough curves at the 0–5 cm sampling depth: (**a**) unamended soil; and (**b**) PET-Fe-BDC composite geotextile-amended soil.

**Figure 15:**
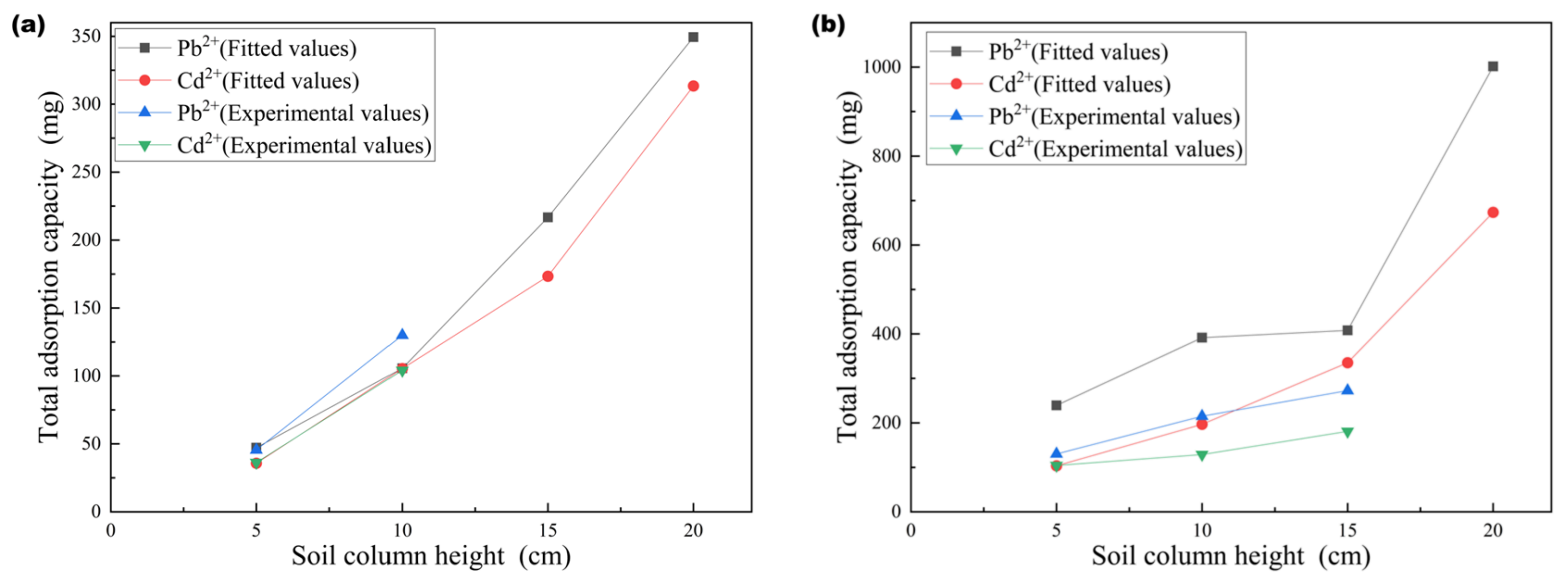
Comparison of the endpoint-integrated experimental qtot values and Thomas-fitted qM parameters at different soil-column depths: (**a**) unamended soil; and (**b**) composite-geotextile-amended soil.

**Figure 16:**
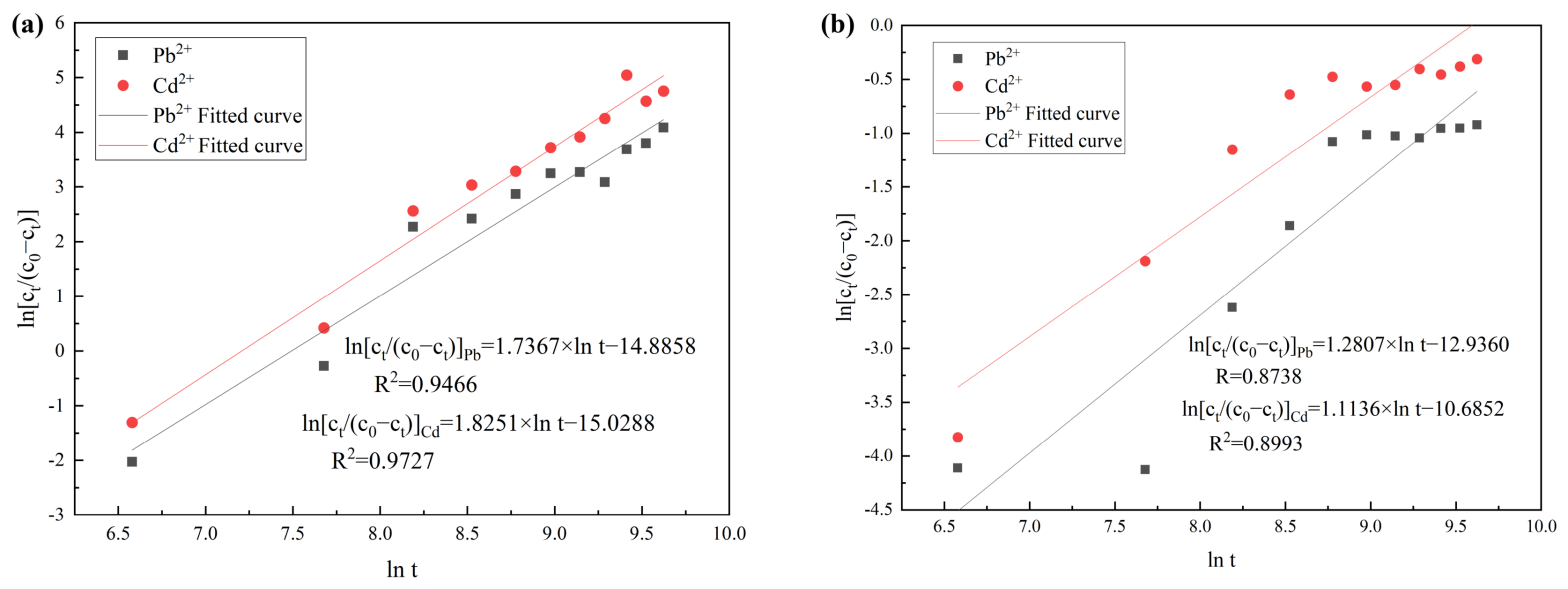
Yan-model fitting of Pb^2+^ and Cd^2+^ breakthrough curves at the 0–5 cm sampling depth: (**a**) unamended soil and (**b**) composite geotextile-amended soil.

**Figure 17:**
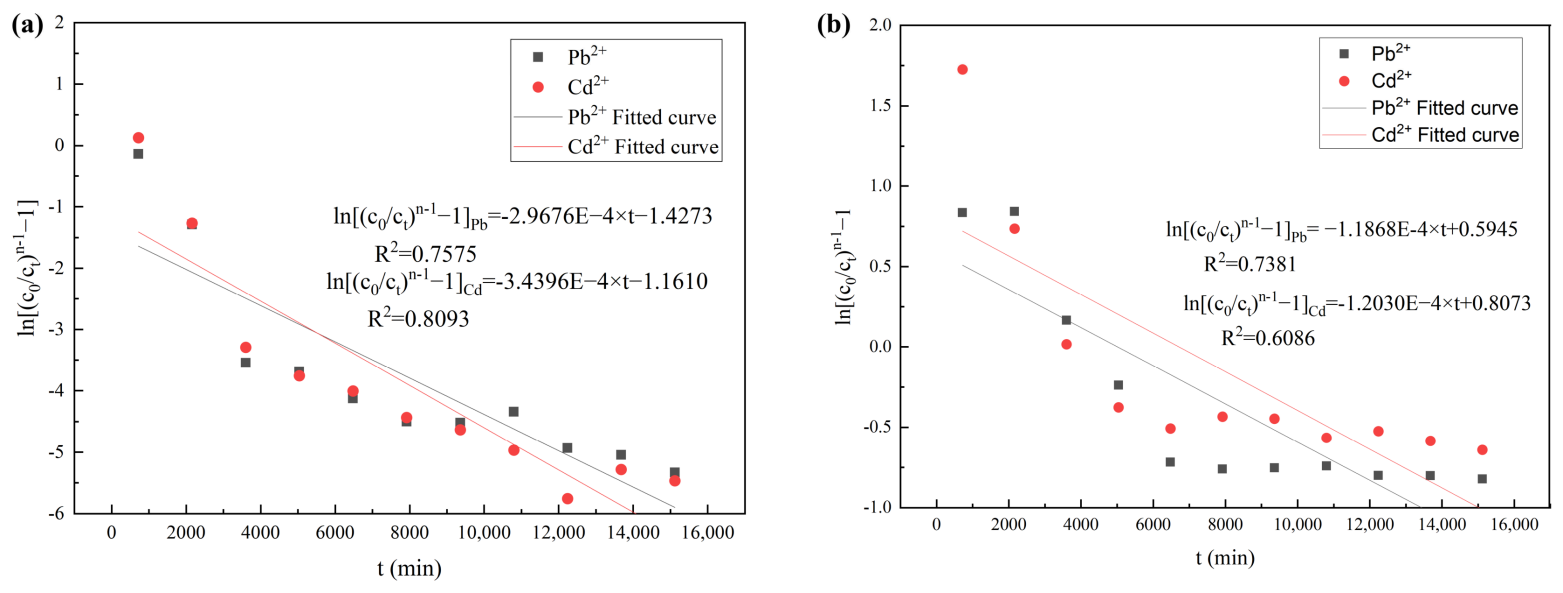
Clark-model fitting of Pb^2+^ and Cd^2+^ breakthrough curves at the 0–5 cm sampling depth: (**a**) unamended soil; and (**b**) composite geotextile-amended soil.

**Figure 18:**
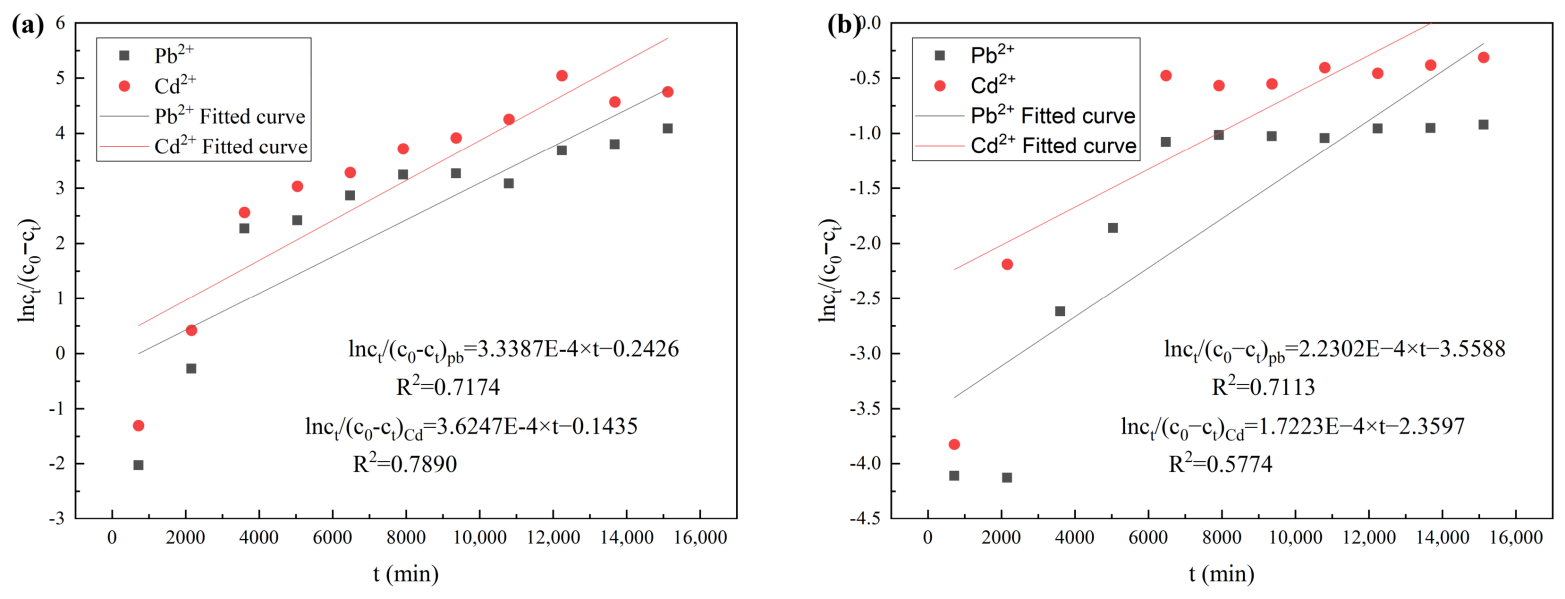
Yoon–Nelson-model fitting of Pb^2+^ and Cd^2+^ breakthrough curves at the 0–5 cm sampling depth: (**a**) unamended soil; and (**b**) composite geotextile-amended soil.

**Table 1:** Basic physical properties of the test soil.

Natural Moisture Content (%)	Liquid Limit (%)	Plastic Limit (%)	Liquidity Index	Plasticity Index	Relative Density of Soil Particles	Permeability Coefficient (cm/s)
33.3	44.29	10.4	0.68	33.85	2.38	1.60 × 10^−5^

**Table 2:** Main properties of the PET geotextile.

Tensile Strength (kN/m^2^)	Elongation at Break (%)	Apparent Opening Size O_90_ (mm)	Vertical Permeability Coefficient (cm/s)	Tear Strength (kN/m^2^)
9.52	63	0.09	3 × 10^−3^	26

**Table 3:** Experimental scheme for soil column leaching tests.

Heavy Metal Species	Concentration (mg/L)	Column Configuration	Flow Rate (mL/h)	Duration (h)	Sampling Port Height (cm)
Combined contamination	20 mg/L Pb^2+^ + 20 mg/L Cd^2+^	Soil only	60	252	5, 10, 15, and 20
		Composite-geotextile-amended soil			

**Table 4:** Dynamic retention parameters of Pb^2+^ and Cd^2+^ for different percolation paths.

Soil Leaching Column	Bed Depth	Heavy Metal Ions	*t_b_* (min)	*t_e_/t_e_*′ (min)	*V_eff_* (mL)	*q_tot_* (mg)	*m_tot_* (mg)	*Y* (%)
Unamended soil	0–5 cm	Pb^2+^	621.72	4617.20	4617.20	45.4759	92.3440	49.25
		Cd^2+^	337.75	3586.01	3586.01	36.2759	71.7202	50.58
	0–10 cm	Pb^2+^	2346.27	13,221.39	13,221.39	103.8226	264.4278	39.26
		Cd^2+^	1745.59	8393.45	8393.45	74.6147	167.8690	44.45
	0–15 cm	Pb^2+^	3857.71	—	—	—	—	—
		Cd^2+^	2603.84	—	—	—	—	—
	0–20 cm	Pb^2+^	8563.14	-	-	-	-	-
		Cd^2+^	5448.12	-	-	-	-	-
Composite geotextile-amended soil	0–5 cm	Pb^2+^	4294.51	7200	7200	130.0126	144	90.29
		Cd^2+^	2146.75	6480	6480	104.0728	129.6	80.30
	0–10 cm	Pb^2+^	6060.96	12,240	12,240	214.8952	244.8	87.78
		Cd^2+^	3197.50	7920	7920	128.8051	158.4	81.32
	0–15 cm	Pb^2+^	9279.10	15,120	15,120	272.6921	302.4	90.18
		Cd^2+^	5138.06	10,800	10,800	180.9958	216	83.79
	0–20 cm	Pb^2+^	—	—	—	—	—	—
		Cd^2+^	—	—	—	—	—	—

An em dash indicates that the corresponding endpoint was not reached within the experimental period. For the composite-system curves, *t_e_*′ denotes the transition point into the slow-retention stage.

## Data Availability

The original contributions presented in this study are included in the article/Supplementary Materials. Further inquiries can be directed to the corresponding author.

## Supplementary Materials

The following supporting information can be downloaded at: https://www.mdpi.com/article/10.3390/ma19194100/s1, Figure S1: Temporal variation of *c_0_*− *c*_t_ in the 0–5 cm soil layer: (a) Pb^2+^ in unamended soil; (b) Cd^2+^ in unamended soil; (c) Pb^2+^ in composite geotextile-amended soil; and (d) Cd^2+^ in composite geotextile-amended soil. Shaded regions identify the integration intervals used to calculate *q*_tot_; Figure S2: Temporal variation of *c_0_*− *c*_t_ in the 0–10 cm soil layer: (a) Pb^2+^ in unamended soil; (b) Cd^2+^ in unamended soil; (c) Pb^2+^ in composite geotextile-amended soil; and (d) Cd^2+^ in composite geotextile-amended soil. Shaded regions identify the integration intervals used to calculate *q*_tot_; Figure S3: Temporal variation of *c_0_*− *c*_t_ in the 0–15 cm soil layer: (a) Pb^2+^ in unamended soil; (b) Cd^2+^ in unamended soil; (c) Pb^2+^ in composite geotextile-amended soil; and (d) Cd^2+^ in composite geotextile-amended soil. Shaded regions identify the integration intervals used to calculate *q*_tot_ where the selected endpoint was reached; Figure S4: Temporal variation of *c_0_*− *c*_t_ in the 0–20 cm soil layer: (a) Pb^2+^ in unamended soil; (b) Cd^2+^ in unamended soil; (c) Pb^2+^ in composite geotextile-amended soil; and (d) Cd^2+^ in composite geotextile-amended soil. Profiles are shown for completeness; endpoint-based quantities are reported only where the selected endpoint was reached; Figure S5: Thomas-model fitting results for Pb^2+^ and Cd^2+^: (a) 0–10 cm, unamended soil; (b) 0–10 cm, composite geotextile-amended soil; (c) 0–15 cm, unamended soil; (d) 0–15 cm, composite geotextile-amended soil; (e) 0–20 cm, unamended soil; and (f) 0–20 cm, composite geotextile-amended soil; Table S1: Thomas-model fitting parameters for the investigated percolation paths; Table S2: Yan-model fitting parameters for the 0–5 cm soil layer; Table S3: Clark-model fitting parameters for the 0–5 cm soil layer; Table S4: Yoon–Nelson-model fitting parameters for the 0–5 cm soil layer; Note S1: Calculation of Dynamic Retention Parameters; Note S2: Breakthrough-Model Formulations and Fitting Parameters.
