# Supplementary material for: PET-Fe-BDC Composite Geotextiles for Groundwater Remediation: In Situ Synthesis and Dynamic Retention of Pb(II) and Cd(II)
Source: Materials (Basel). 2026 Sep 25;19(19):4100. doi: 10.3390/ma19194100 (PMC13666042; doi:10.3390/ma19194100)
Supplement: Supplementary file 1 [file materials-19-04100-s001.zip › materials-4482462-supplementary.pdf]

Supplementary Information

PET–Fe-BDC Composite Geotextiles for Groundwater Remediation: In Situ Synthesis and Dynamic Retention of Pb(II) and Cd(II)

Li Shao, Kang Wu, Jing Ni, Jiawei Xu, Tongtong Wang, Jili Qu, and Chao Bao

Supplementary Note S1. Calculation of Dynamic Retention Parameters

Dynamic retention parameters were calculated from the concentration-deficit curve (c_0-c_t) as a function of time. Formal breakthrough was defined at c_0/c_t = 0.10. The endpoint t_e denotes the selected saturation endpoint; for composite-system curves that entered a stable slow-retention stage within the experimental period, t_e' was used as the operational evaluation endpoint. The calculations were performed using the following relationships.

$$V_{eff} = Q.t_e \tag{S1}$$

$$q_{tot} = \frac{Q.A}{1000} = \frac{Q}{1000}\int_{t=0}^{t=t_e} (c_0 - c_t)d_t \tag{S2}$$

$$m_{tot} = \frac{c_0.Q.t_e}{1000} \tag{S3}$$

$$Y = \frac{q_{tot}}{m_{tot}} \times 100\% \tag{S4}$$

Here, Q is the influent flow rate (mL/min); c_0 and c_t are the influent concentration and the concentration measured at time t (mg/L), respectively; A is the integrated area under the (c_0-c_t)-versus-time curve; V_{eff} is the cumulative influent volume (mL); q_{tot} is the cumulative retained mass (mg); m_{tot}, is the cumulative influent mass (mg); and Y is the cumulative removal efficiency (%). The parameters t_b, t_e, and t_e' denote the formal breakthrough time, the selected saturation endpoint, and the transition point into the slow-retention stage, respectively. The resulting values are summarized in Table 4 of the main manuscript.

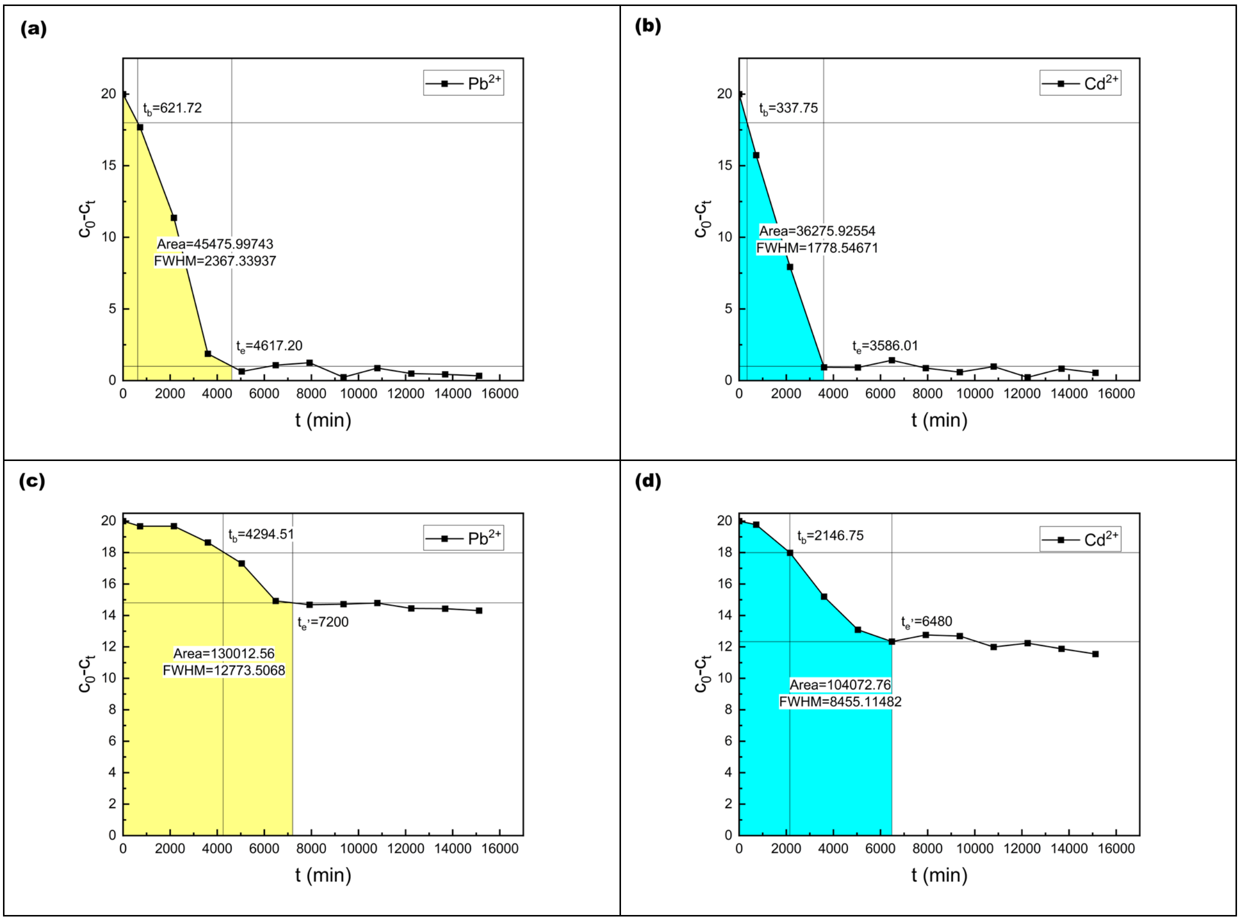

Figure S1. Temporal variationof c_0-c_t in the 0–5 cm soil layer: (a) Pb^{2+} in unamended soil; (b) Cd^{2+} in unamended soil; (c) Pb^{2+} in composite geotextile-amended soil; and (d) Cd^{2+} in composite geotextile-amended soil. Shaded regions identify the integration intervals used to calculate q_{tot}.

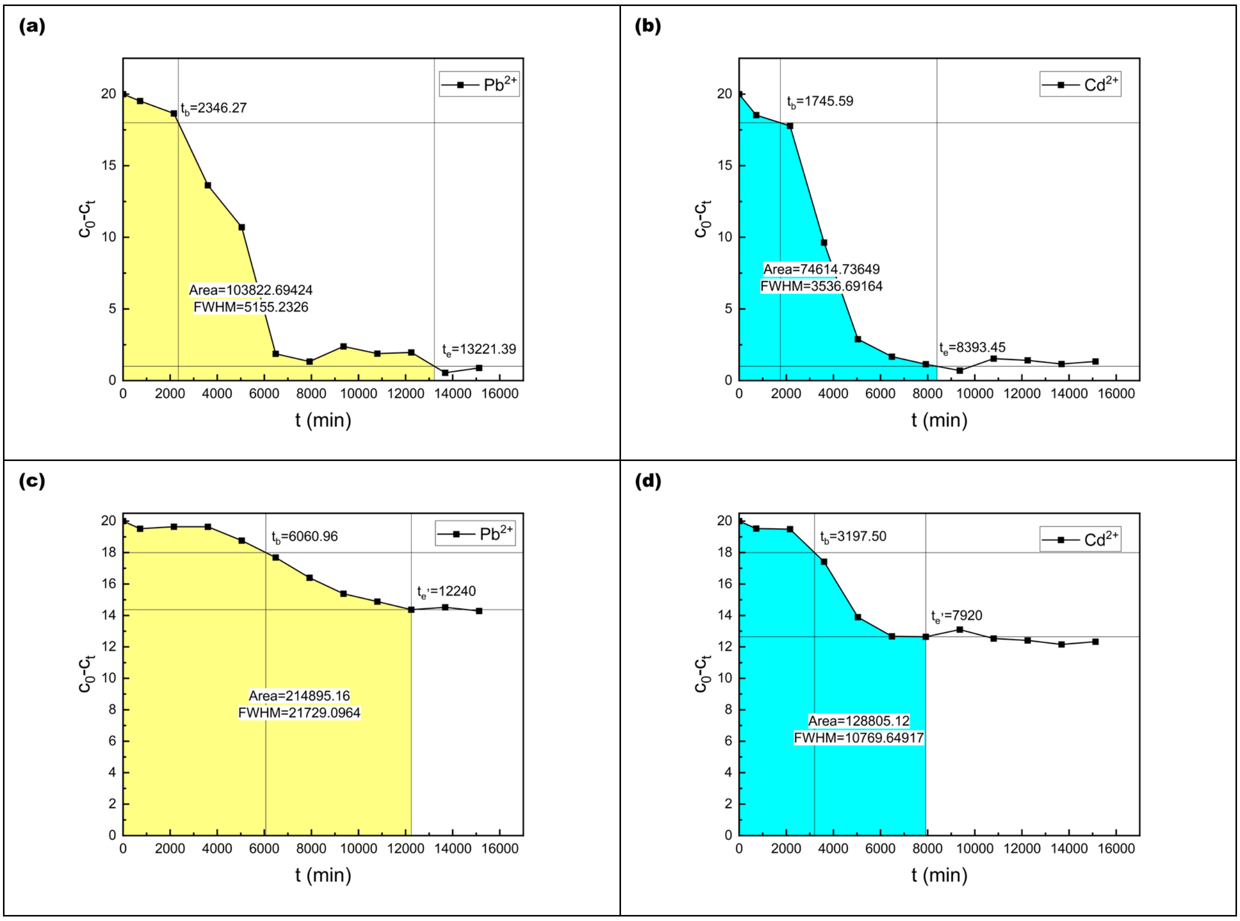

Figure S2. Temporal variation of c_0-c_t in the 0–10 cm soil layer: (a) Pb^{2+} in unamended soil; (b) Cd^{2+} in unamended soil; (c) Pb^{2+} in composite geotextile-amended soil; and (d) Cd^{2+} in composite geotextile-amended soil. Shaded regions identify the integration intervals used to calculate q_{tot}.

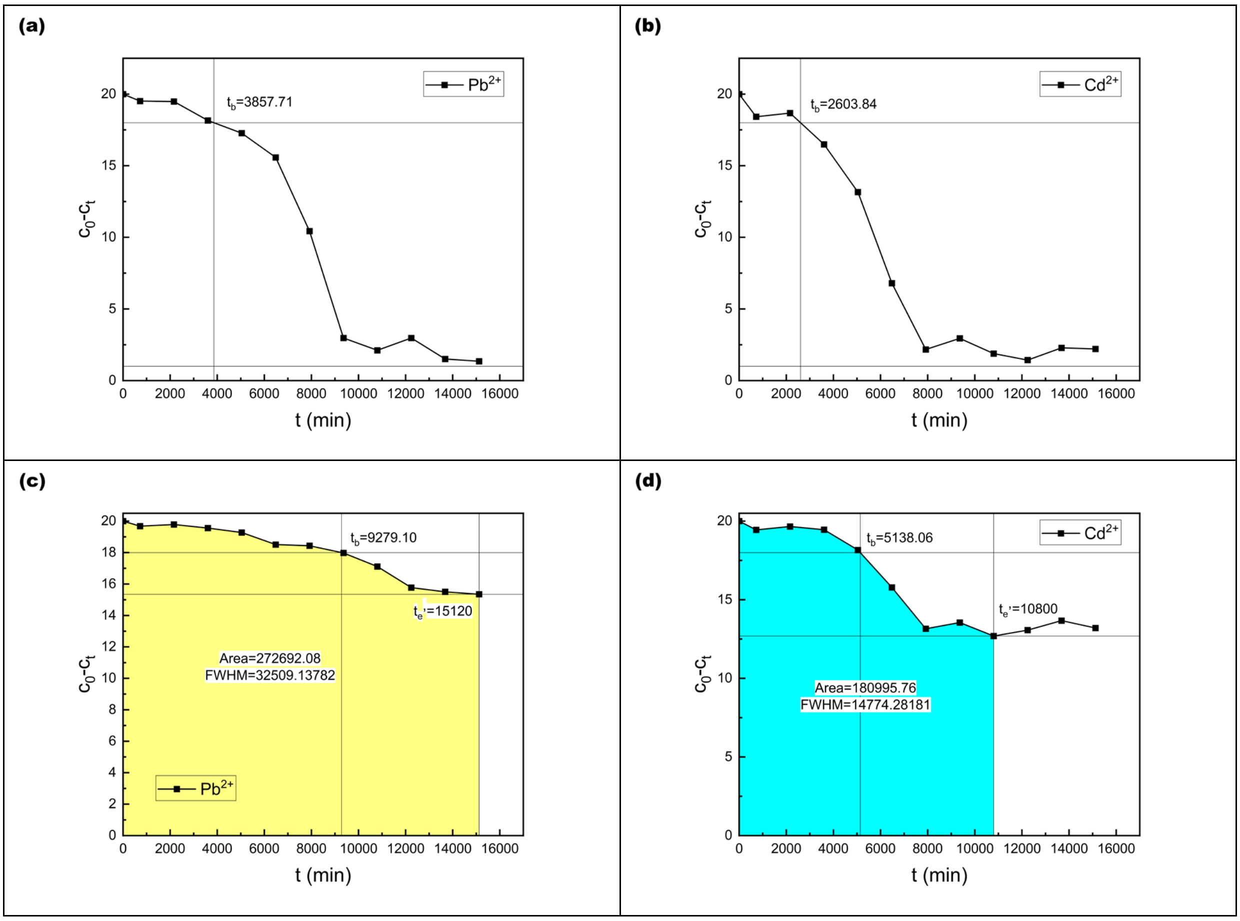

Figure S3. Temporal variation of c_0-c_t in the 0–15 cm soil layer: (a) Pb^{2+} in unamended soil; (b) Cd^{2+} in unamended soil; (c) Pb^{2+} in composite geotextile-amended soil; and (d) Cd^{2+} in composite geotextile-amended soil. Shaded regions identify the integration intervals used to calculate q_{tot} where the selected endpoint was reached.

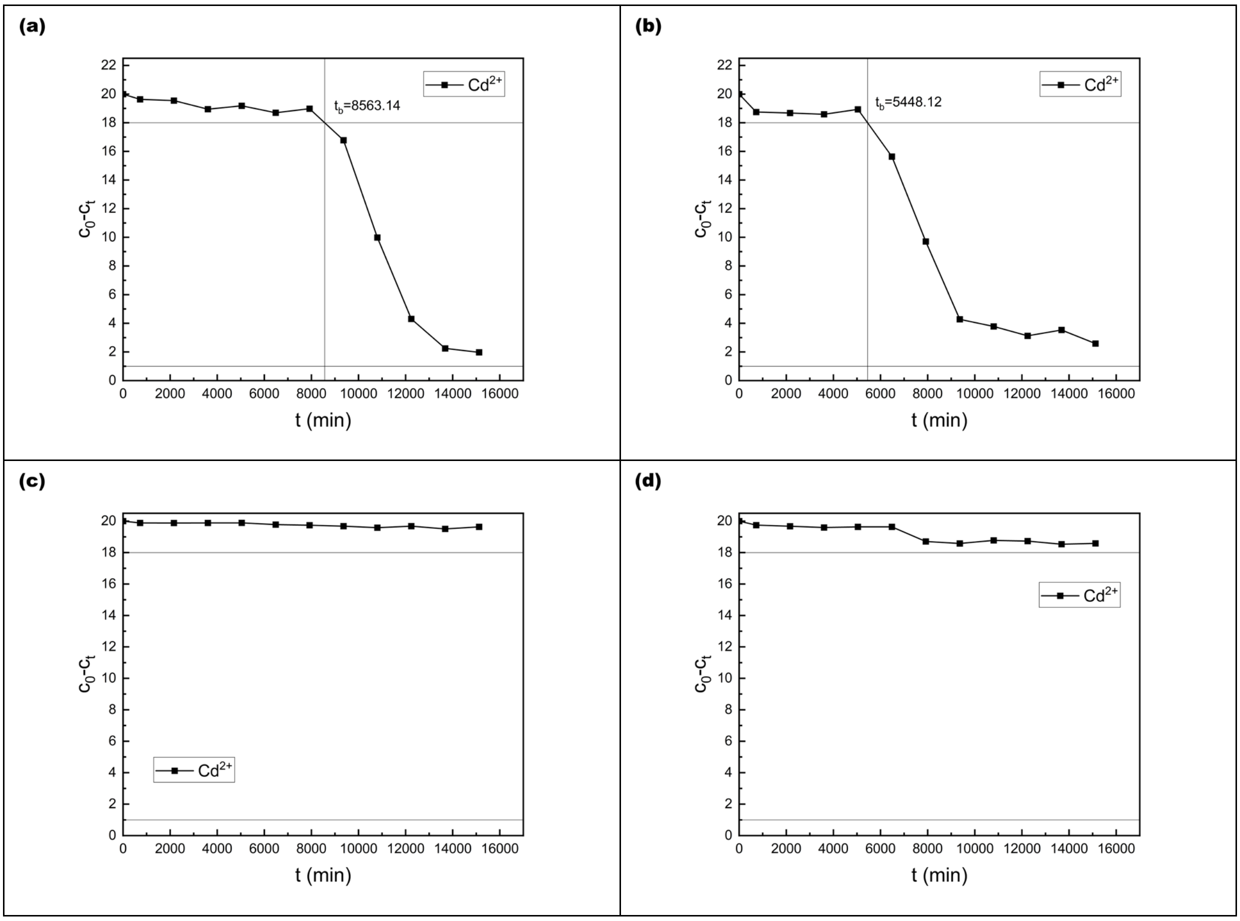

Figure S4. Temporal variation of c_0-c_t in the 0–20 cm soil layer: (a) Pb^{2+} in unamended soil; (b) Cd^{2+} in unamended soil; (c) Pb^{2+} in composite geotextile-amended soil; and (d) Cd^{2+} in composite geotextile-amended soil. Profiles are shown for completeness; endpoint-based quantities are reported only where the selected endpoint was reached.

Supplementary Note S2. Breakthrough-Model Formulations and Fitting Parameters

The Thomas, Yan, Clark, and Yoon–Nelson models were used to compare the measured breakthrough behavior. Model parameters were obtained from the same concentration-time datasets used in the main manuscript. The model equations and parameter definitions are provided below, followed by the complete fitting results.

S2.1. Thomas Model

The Thomas model and its linearized form were expressed as:

$$\frac{c_t}{c_0} = \frac{1}{1 + exp\left[\frac{K_{Th}}{Q} qM - K_{Th} c_0 t\right]} \tag{S5}$$

$$\frac{c_t}{c_0} - 1 = 1 + exp\left[\frac{K_{Th}}{Q} qM - K_{Th} c_0 t\right] \tag{S6}$$

K_{Th} is the Thomas rate constant (mL mg^{-1} min^{-1}), Q is the volumetric flow rate (mL/min), qM is the Thomas-model estimate of the total retained mass (mg), c_0 is the influent concentration (mg/L), c_t is the concentration at time t (mg/L), and t is time (min). Figure S5 presents the complete depth-resolved fitting results, and Table S1 summarizes the corresponding parameters.

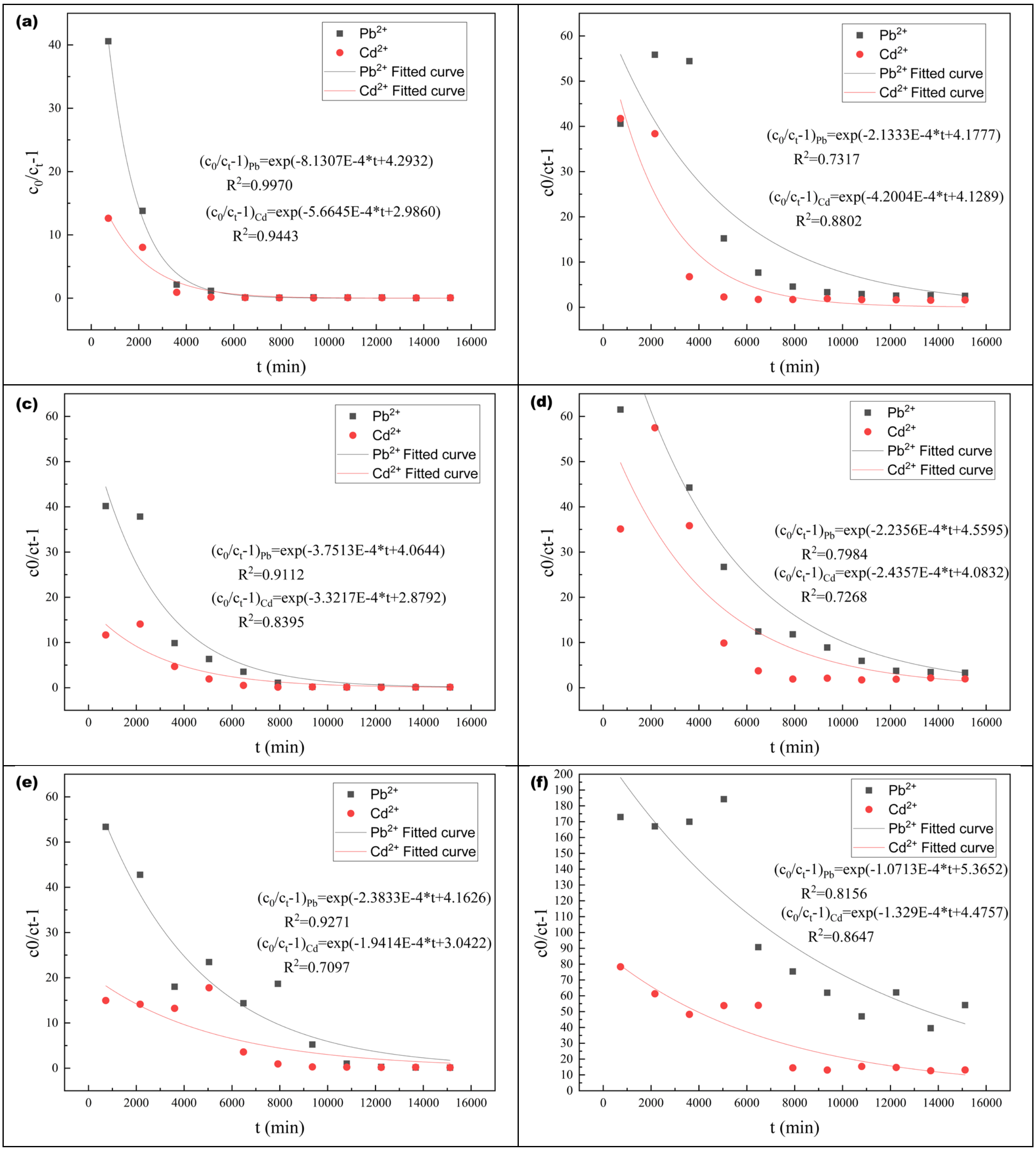

Figure S5. Thomas-model fitting results for Pb^{2+} and Cd^{2+}: (a) 0–10 cm, unamended soil; (b) 0–10 cm, composite geotextile-amended soil; (c) 0–15 cm, unamended soil; and (d) 0–15 cm, composite geotextile-amended soil. (e) 0–20 cm, unamended soil; and (f) 0–20 cm, composite geotextile-amended soil.

Table S1. Thomas-model fitting parameters for the investigated percolation paths.

Column system	Depth (cm)	Ion	K_{Th} (mL mg⁻¹ min⁻¹)	qM (mg)	Experimental q_{tot} (mg)	R^2
Unamended soil	0–5	Pb^{2+}	0.06150	47.1984	45.4779	0.9995
Unamended soil	0–5	Cd^{2+}	0.06200	35.7721	36.2772	0.9983
Unamended soil	0–10	Pb^{2+}	0.04065	105.6047	103.8226	0.9970
Unamended soil	0–10	Cd^{2+}	0.02832	105.4285	74.6147	0.7317
Unamended soil	0–15	Pb^{2+}	0.01876	216.6929	—	0.9112
Unamended soil	0–15	Cd^{2+}	0.01661	173.3570	—	0.7984
Unamended soil	0–20	Pb^{2+}	0.01192	349.3140	—	0.9271
Unamended soil	0–20	Cd^{2+}	0.00971	313.4027	—	0.8156
Composite geotextile-amended soil	0–5	Pb^{2+}	0.01881	239.3036	130.0126	0.8667
Composite geotextile-amended soil	0–5	Cd^{2+}	0.05600	103.2625	104.0728	0.9999
Composite geotextile-amended soil	0–10	Pb^{2+}	0.01067	391.6655	214.8952	0.9443
Composite geotextile-amended soil	0–10	Cd^{2+}	0.02100	196.6143	128.8051	0.8802
Composite geotextile-amended soil	0–15	Pb^{2+}	0.01118	407.8994	272.6921	0.8395
Composite geotextile-amended soil	0–15	Cd^{2+}	0.01218	335.2794	180.9958	0.7268
Composite geotextile-amended soil	0–20	Pb^{2+}	0.005357	1001.624	—	0.7097
Composite geotextile-amended soil	0–20	Cd^{2+}	0.006645	673.544	—	0.8647

An em dash indicates that an endpoint-based experimental q_{tot} value was not assigned for that curve within the selected evaluation interval.

Note: Experimental q_{tot} was obtained by numerical integration up to the selected evaluation endpoint, whereas qM is a Thomas-model fitting parameter. For curves that did not reach complete breakthrough, qM represents an extrapolated model parameter rather than an experimentally verified total retention capacity.

S2.2. Yan Model

The Yan dose-response model and its linearized form were expressed as:

$$\frac{c_t}{c_0} = 1 - \frac{1}{1 + \left(\frac{Q^2 t}{k_y q_y m}\right)^{\frac{k_y c_0}{Q}}} \quad \text{(S7)}$$

$$\ln\left(\frac{c_t}{c_0 - c_t}\right) = \left(\frac{k_y c_0}{Q}\right)\ln\left(\frac{Q^2}{k_y q_y m}\right) + \left(\frac{k_y c_0}{Q}\right)\ln t \quad \text{(S8)}$$

Here, k_y is the Yan rate constant (L mg⁻¹ min⁻¹), q_y is the maximum adsorption capacity estimated by the Yan model (mg/g), m is the adsorbent mass (g), and Q is the volumetric flow rate (L/min). The 0–5 cm fitting curves are presented in Figure 16 of the main manuscript; Table S2 provides the complete parameter set.

Table S2. Yan-model fitting parameters for the 0–5 cm soil layer.

Column system	Ion	k_y (L mg⁻¹ min⁻¹)	q_y m (mg)	Experimental q (mg)	R^2
Unamended soil	Pb^{2+}	8.6835E−5	60.7826	45.4779	0.9466
Unamended soil	Cd^{2+}	9.1255E−5	41.2995	36.2772	0.9727
Composite geotextile-amended soil	Pb^{2+}	6.4035E−5	380.4278	130.0126	0.8738
Composite geotextile-amended soil	Cd^{2+}	5.5680E−5	263.8988	104.0728	0.8933

S2.3. Clark Model

The Clark model and its linearized form were expressed as:

$$\frac{c_t}{c_0} = \left(\frac{1}{1 + A\mathrm{e}^{-rt}}\right)^{\frac{1}{n-1}} \tag{S9}$$

$$\ln\left[\left(\frac{c_0}{c_t}\right)^{n-1} - 1\right] = \ln A - rt \tag{S10}$$

Here, n is the Freundlich isotherm coefficient, and A and r are parameters of the breakthrough-curve equation. Figure S6 and Table S3 provide the 0–5 cm fitting results and parameter values.

Table S3. Clark-model fitting parameters for the 0–5 cm soil layer.

Column system	Ion	n	A	r (min⁻¹)	R²
Unamended soil	Pb^{2+}	1.2895	0.2400	2.9676E−4	0.7575
Unamended soil	Cd^{2+}	1.4914	0.3132	3.4396E−4	0.8093
Composite geotextile-amended soil	Pb^{2+}	1.2895	1.8121	1.1868E−4	0.7381
Composite geotextile-amended soil	Cd^{2+}	1.4914	2.2418	1.2030E−4	0.6086

S2.4. Yoon–Nelson Model

The linear form of the Yoon–Nelson model was expressed as:

$$\ln\left(\frac{c_t}{c_0 - c_t}\right) = k_{YN}t - k_{YN}\tau \tag{S11}$$

K_{YN} is the Yoon–Nelson rate constant (min⁻¹), and τ is the fitted characteristic time parameter (min). Figure S7 and Table S4 provide the 0–5 cm fitting results and parameter values.

Table S4. Yoon–Nelson-model fitting parameters for the 0–5 cm soil layer.

Column system	Ion	K_{YN} (min⁻¹)	τ(min)	$t_{0.5}$ (min)	R^2
Unamended soil	Pb^{2+}	3.3387E−4	716.23	2366.05	0.7174
Unamended soil	Cd^{2+}	3.6247E−4	395.89	1778.16	0.7890
Composite geotextile-amended soil	Pb^{2+}	2.2302E−4	15944	—	0.7113
Composite geotextile-amended soil	Cd^{2+}	1.7223E−4	13700	—	0.5774
